# Transmembrane signal transduction by peptide hormones via family B G protein-coupled receptors

**DOI:** 10.3389/fphar.2015.00264

**Published:** 2015-11-05

**Authors:** Kelly J. Culhane, Yuting Liu, Yingying Cai, Elsa C. Y. Yan

**Affiliations:** ^1^Department of Molecular Biophysics and Biochemistry, Yale UniversityNew Haven, CT, USA; ^2^Department of Chemistry, Yale UniversityNew Haven, CT, USA

**Keywords:** GPCR, family B GPCR, peptide hormone, G protein, activation mechanisms, signal transduction

## Abstract

Although family B G protein-coupled receptors (GPCRs) contain only 15 members, they play key roles in transmembrane signal transduction of hormones. Family B GPCRs are drug targets for developing therapeutics for diseases ranging from metabolic to neurological disorders. Despite their importance, the molecular mechanism of activation of family B GPCRs remains largely unexplored due to the challenges in expression and purification of functional receptors to the quantity for biophysical characterization. Currently, there is no crystal structure available of a full-length family B GPCR. However, structures of key domains, including the extracellular ligand binding regions and seven-helical transmembrane regions, have been solved by X-ray crystallography and NMR, providing insights into the mechanisms of ligand recognition and selectivity, and helical arrangements within the cell membrane. Moreover, biophysical and biochemical methods have been used to explore functions, key residues for signaling, and the kinetics and dynamics of signaling processes. This review summarizes the current knowledge of the signal transduction mechanism of family B GPCRs at the molecular level and comments on the challenges and outlook for mechanistic studies of family B GPCRs.

## Introduction

**Figure 1:**
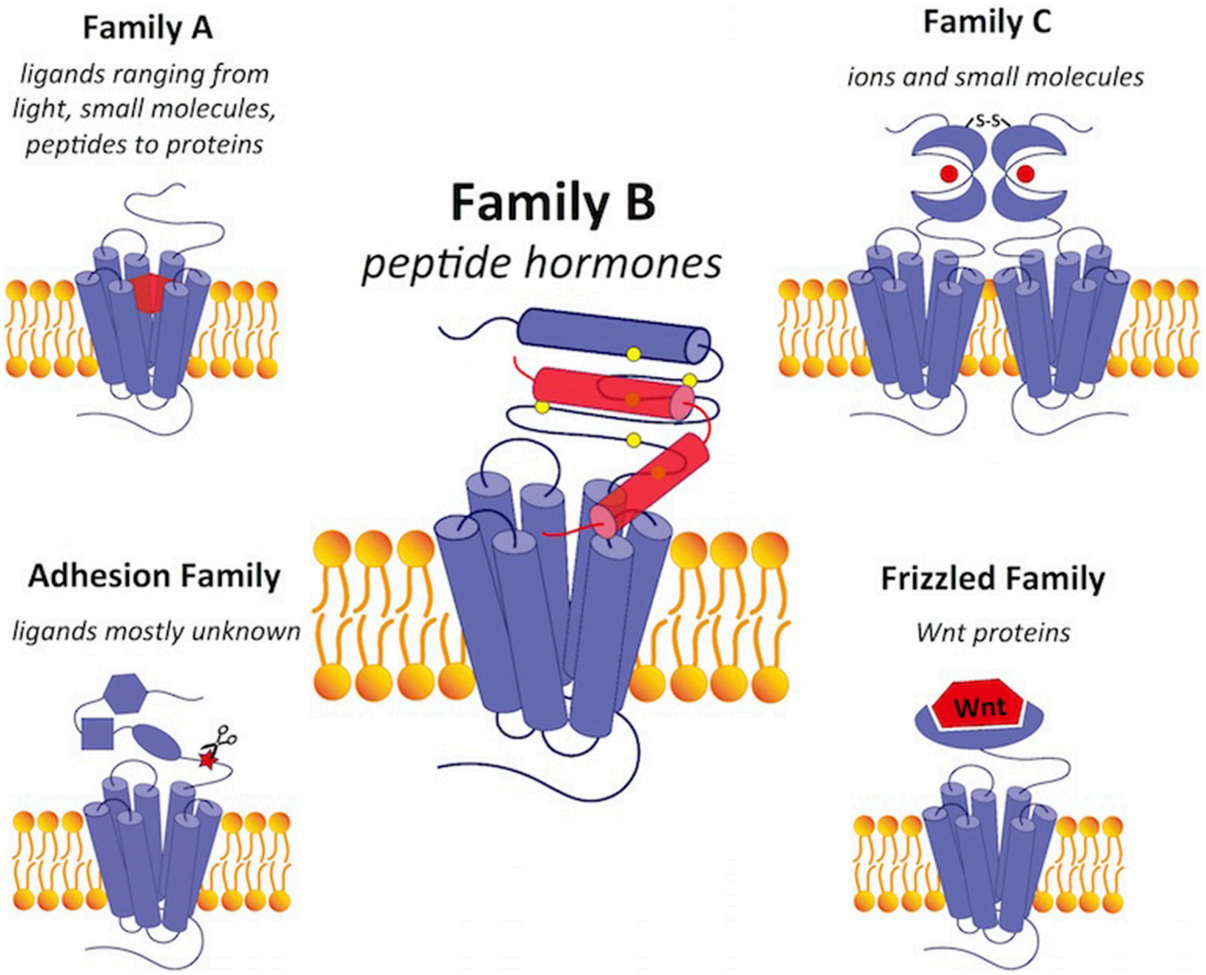
**A comparison of GPCRs in family B with GPCRs in the other four families**. Family B GPCRs detect peptide hormones and have a relatively large extracellular N-terminus (~120 amino acids) with a conserved structural fold stabilized by cysteine bonds. Family A GPCRs bind a wide range of diverse ligands in the transmembrane region and have a small extracellular domain. Family C receptors have a large extracellular N-terminal ligand-binding region in the “Venus flytrap” fold for ligand binding with a conserved disulfide linkage to form a dimer. The adhesion and frizzled families have GPCR-like transmembrane-spanning regions fused together with one or several functional N-terminal domains. Ligands are shown in red. Scissors in the adhesion family indicate the autoproteolysis-inducing domain.

G protein-coupled receptors (GPCRs), which form the largest protein superfamily of the vertebrate genome, play an important role in signal transduction by detecting extracellular stimuli and activating intracellular downstream pathways (Figure 1; Fredriksson et al., 2003). All GPCRs share a common seven-transmembrane topology, and mediate cellular responses through interactions with a variety of extracellular signals. These extracellular signals range from photons and small molecules to hormones and proteins, indicating the structural and functional diversity of over 800 different GPCRs (Lagerstrom and Schioth, 2008). Despite the diversity, GPCRs share a general mechanism of activation that begins with ligand binding, which causes conformational rearrangements in the seven-transmembrane (7TM) domain that activate the G protein in the cytoplasmic region. G protein activation triggers various signaling cascades to mediate physiological processes. Since the ligand binding sites are highly specific, GPCRs have been heavily exploited as drug targets. They are the targets for over 40% of the current pharmaceutical drugs on the market with an estimated global sales of ~$85 billion (Stevens et al., 2013).

Although most drugs developed against GPCRs target family A receptors, family B GPCRs are becoming increasingly attractive drug targets, especially for treatment of metabolic diseases (Tautermann, 2014). The 15 family B GPCRs for various peptide hormones are grouped into subfamilies based on their physiological roles, including insulin secretion, vasodilation, regulation of Ca^2+^ homeostasis, and cardiac contractility (Table 1). Nearly all family B GPCRs have been validated as drug targets for diseases, such as cancer, osteoporosis, diabetes, cardiovascular disease, neurodegeneration, and migraine (Table 1; Archbold et al., 2011). However, it has been challenging to identify small-molecule therapeutics to target this family, with only a few peptide agonists approved by the FDA as drugs. Most of these agonist drugs are derivatives of the family B cognate peptide ligands (Table 1). The challenge of identifying conventional small-molecule drugs lies in the lack of molecular information to locate druggable binding sites for *in silico* drug design and screening. Nevertheless, the recently reported and first available crystal structures of the transmembrane domain (TMD) of two family B GPCRs are expected to advance the development (Hollenstein et al., 2013; Siu et al., 2013).

**Table 1:** **Summary of Family B GPCR physiology and drugs (Hoare, 2005; Archbold et al., 2011; Bortolato et al., 2014)**.

**Receptor(s)**	**Ligands**	**Physiological functions**	**Disease(s)**	**Available drugs**
**CALCITONIN RECEPTOR FAMILY**				
Calcitonin receptor (CTR)	Calcitonin	Ca^2+^ homeostasis	Osteoporosis	Miacalcin, Fortical
Amylin receptors (AMY_1_, AMY_2_, AMY_3_)	Amylin	Energy homeostasis and body fluid balance	Diabetes/obesity	Pramlintide
	Amyloid-beta (Aβ) protein			
Calcitonin gene-related peptide receptor (CGRP receptor)	α-calcitonin gene-related peptide (α-CGRP)	Vasodilation and nociception	Migraine	
	ß-CGRP			
Adrenomedullin receptors (AM_1_, AM_2_)	adrenomedullin 1	Vasodilation	Cardiovascular disease, cancer	
	adrenomedullin 2			
**GLUCAGON RECEPTOR FAMILY**				
Glucagon receptor (GCGR)	Glucagon	Regulation of blood glucose	Diabetes	Glucagon
Glucagon-like peptide 1 receptor (GLP-1R)	Glucagon-like peptide 1 (GLP-1)	Insulin and glucagon secretion	Diabetes	Exenatide, Lixisenatide, Liraglutide, Albiglutide, Dulaglutide
Glucagon-like peptide 2 receptor (GLP-2R)	Glucagon-like peptide 2 (GLP-2)	Gut mucosal growth	Short bowel syndrome, Crohn's disease, osteoporosis	Teduglutide
Gastric inhibitory polypeptide receptor (GIPR)	Gastric inhibitory polypeptide (GIP)	Insulin secretion, fatty acid metabolism	Diabetes/obesity	
**CORTICOTROPIN-RELEASING HORMONE RECEPTORS**				
Corticotropin-releasing factor receptor 1 (CRF1R)	Corticotropin-releasing factor (CRF)	Release of ACTH and central stress responses	Stress, inflammatory bowel syndrome	Corticorelin
	Urocortin I (Ucn1)			
Corticotropin-releasing factor receptor 2 (CRF2R)	CRF	Central stress responses, cardiac contractility, hearing	Cancer, heart failure, hypertension	
	Urocortin II (Ucn2)			
	Urocortin III (Ucn3)			
**PARATHYROID HORMONE RECEPTORS**				
Parathyroid hormone 1 receptor (PTH1R)	Parathyroid hormone (PTH)	Ca^2+^ homeostasis	Osteoporosis, hypoparathyroidism	Teriparatide, Preotact
	Parathyroid hormone-related protein (PTHrP)	Developmental regulator		
Parathyroid hormone 2 receptor (PTH2R)	PTH	Hypothalamic secretion, nociception	Nociception	
	Tuberoinfundibular peptide of 39 residues (TIP39)			
**VASOACTIVE INTESTINAL POLYPEPTIDE RECEPTORS**				
Vasoactive intestinal polypeptide receptor 1 (VPAC_1_R)	Vasoactive intestinal polypeptide (VIP)	Vasodilation, neurotransmission, neuroendocrine functions	Inflammation, neurodegeneration	
	PACAP			
Vasoactive intestinal polypeptide receptor 2 (VPAC_2_R)	VIP	Vasodilation, neurotransmission neuroendocrine functions	Inflammation, neurodegeneration	
	PACAP			
**OTHERS**				
Pituitary adenylate cyclase-activating polypeptide type I receptor (PAC1R)	Pituitary adenylate cyclase-activating polypeptide (PACAP)	Neurotransmission neuroendocrine functions	Neurodegeneration nociception, glucose homeostasis	
Growth-hormone-releasing hormone receptor (GHRHR)	Growth hormone-releasing hormone (GRHR)	Release of growth hormone	Dwarfism, HIV-related lipodystrophy	Tesamorelin, Sermorelin, CJC-1295
Secretin receptor (SCTR)	Secretin	Pancreatic secretion	Autism, schizophrenia, duodenal ulcers, gastrinoma	

Compared to GPCRs in other families (Figure 1), family B GPCRs have a relatively large N-terminus that is ~120 amino acids long. Their N-terminus shares a similar fold stabilized by three conserved disulfide bridges, which construct part of the binding site for ligand recognition. While GPCRs in other families have a wide range of ligands, such as small molecules, ions, lipids, and proteins, all family B receptors bind peptide hormones, modulating physiological processes such as calcium homeostasis and regulation of blood glucose, as summarized in Table 1.

**Figure 2:**
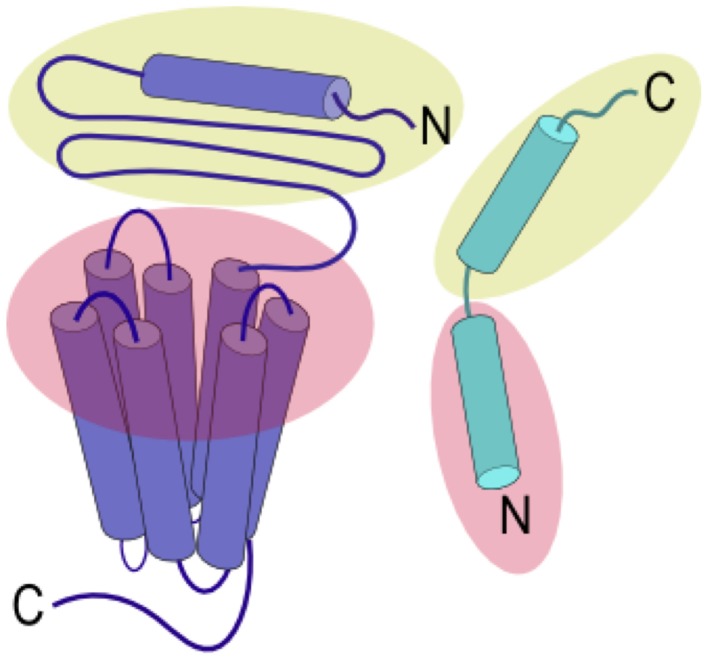
**Two-domain binding model of family B GPCRs**. The C-terminal region of the peptide ligand interacts with the N-terminal domain of the receptor (yellow), allowing the N-terminus of the peptide ligand to interact with the juxtamembrane and TMD (red) to activate the receptor.

Ligands that activate family B GPCRs are peptide hormones with a length of 26 to 114 amino acids. Most ligands of family B GPCRs lack ordered structure in aqueous solutions but form α-helical fragments upon binding to their cognate receptors or under structure-inducing conditions such as in the presence of organic solvents, lipids, or upon crystallization (as reviewed in Parthier et al., 2009). It is widely accepted that the peptide hormones interact with family B GPCRs following the “two-domain” model, in which the peptide hormone's C-terminus binds to their cognate receptor's N-terminal domain and the N-terminus binds to the receptor's juxtamembrane and transmembrane domains (Figure 2). Such binding leads to conformational changes in the receptor's cytoplasmic domain to active a G protein to trigger a downstream signaling process (Pal et al., 2012).

**Figure 3:**
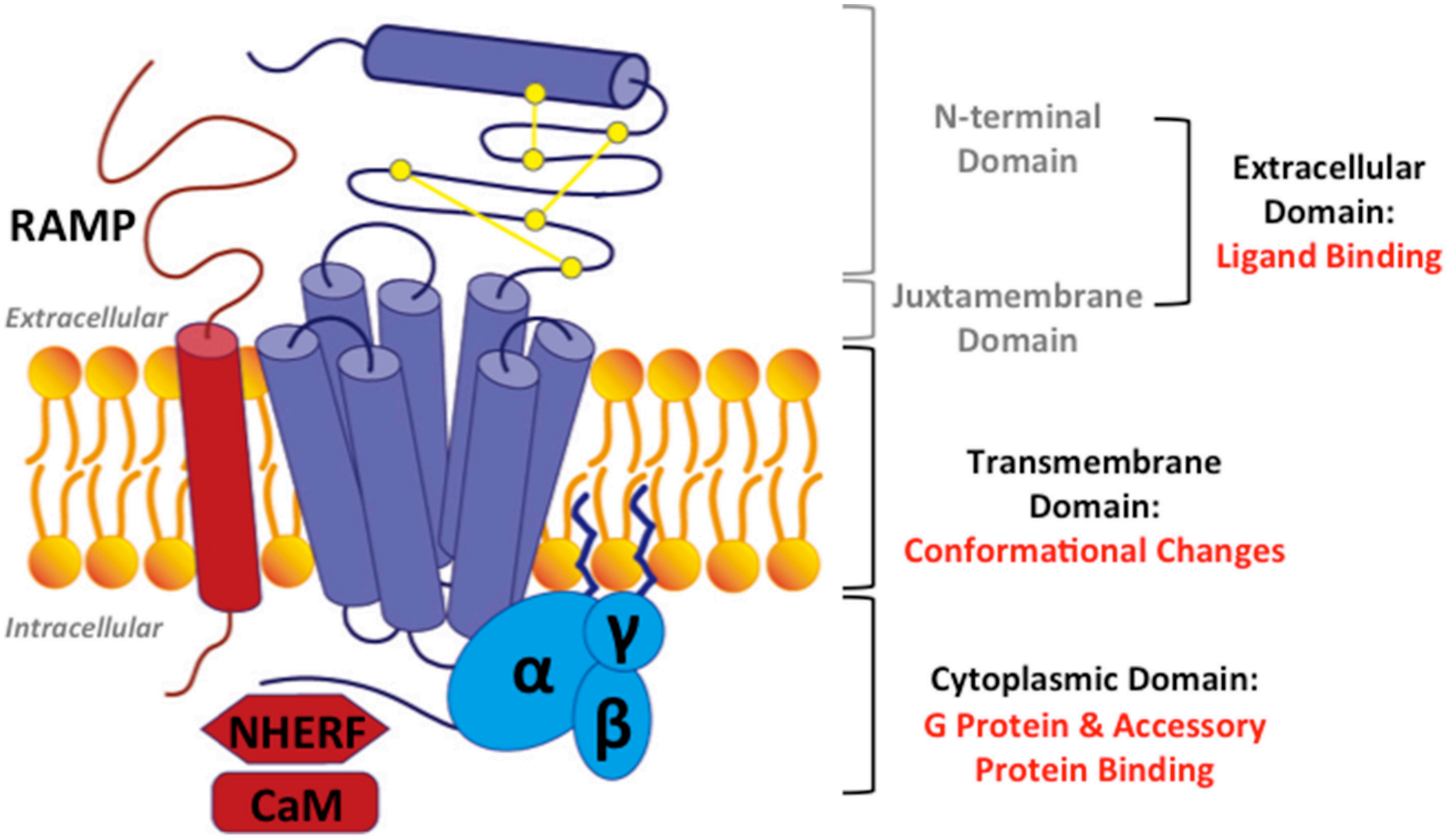
**Structural model for family B GPCRs**. Three domains in family B GPCRs: (1) the extracellular domain, comprising of the N-terminal domain and the juxtamembrane domain, (2) the transmembrane domain (TMD), and (3) the cytoplasmic domain. Three accessory proteins: (1) receptor activity modifying proteins (RAMPs), (2) Na/H exchange regulatory factors (NHERFs), (3) calmodulin (CaM). Purple: a family B GPCR, yellow: three conserved disulfide bonds, blue: a heterotrimeric G protein, and red: accessory proteins.

Tremendous progress has been made in the past 15 years to obtain structural information about GPCRs; however, knowledge about the structure of family B GPCRs is still very limited. Most of the previous structural studies of GPCRs focus on family A receptors, with over a hundred solved crystal structures of over 20 different receptors. For family B GPCRs, most efforts were focused on obtaining the crystal structures of the N-terminal ligand-binding domains (Grace et al., 2007; Parthier et al., 2007; Sun et al., 2007; Pioszak and Xu, 2008; Pioszak et al., 2008, 2009; Runge et al., 2008; Pal et al., 2010; Kumar et al., 2011) until Hollenstein et al. and Siu et al. reported the crystal structures of transmembrane domain of two family B receptors in 2013 (Table 2; Hollenstein et al., 2013; Siu et al., 2013). Although this structural information has provided insights into the structural and functional diversity of family B GPCRs, there is still no full-length structure of family B GPCRs available. Hence, how the extracellular ligand binding domain and transmembrane domain of family B GPCRs cooperate to transduce a signal across the cell membrane upon ligand binding remains largely unknown. Here, we will review the recent progress in addressing this question by surveying the structural, biochemical, and biophysical studies of various domains of family B GPCRs (Figure 3).

**Table 2:** **Structures (X-ray or NMR) available for family B GPCR domains**.

**Receptor(s)**	**Domain**	**PDB ID**	**Ligand used**	**Resolution (Å)**
**CALCITONIN RECEPTOR FAMILY**				
CGRP receptor (ter Haar et al., 2010)	N-terminal LBD complex (CLR 23–133 + RAMP1 26–117)	3N7P	N/A	2.80
	N-terminal LBD complex (CLR 23–133 + RAMP1 26–117)	3N7R	telcagepant (a small molecule antagonist)	2.90
	N-terminal LBD complex (CLR 23–133 + RAMP1 26–117)	3N7S	olcegepant (a small molecule antagonist)	2.10
AM1 receptor (Kusano et al., 2012)	N-terminal LBD complex (CLR 23–136 + RAMP2 39–139)	3AQF	No ligand	2.60
**GLUCAGON RECEPTOR FAMILY**				
Glucagon receptor	N-terminal LBD 28–123	4ERS (Koth et al., 2012)	No ligand	2.64
	N-terminal LBD 29–123	4LF3 (Mukund et al., 2013)	No ligand	2.73
	TMD 123–434	4L6R (Siu et al., 2013)	N/A	3.30
GLP-1R	N-terminal LBD 24–145	3IOL (Underwood et al., 2010)	GLP-1(7–37)	2.10
	N-terminal LBD complex 24–145	3C5T (Runge et al., 2008)	Exendin-4(9–39)	2.10
	N-terminal LBD 24–145	3C59 (Runge et al., 2008)	(SeMet14,21)-exendin-4(9–39)	2.30
GIPR	N-terminal LBD 24–138	4HJ0 (Ravn et al., 2013)	Antibody	3.00
	N-terminal LBD 29–138	2QKH (Parthier et al., 2007)	GIP (1–42)	1.90
**CORTICOTROPIN-RELEASING HORMONE RECEPTORS**				
CRF1R	N-terminal LBD 24–119	3EHS (Pioszak et al., 2008)	N/A	2.76
	N-terminal LBD 24–119	3EHT (Pioszak et al., 2008)	CRF27–41	3.40
	N-terminal LBD 24–119	3EHU (Pioszak et al., 2008)	CRF22–41	1.96
	TMD 104–373	4K5Y (Hollenstein et al., 2013)	Antagonist: CP-376395 (small molecule)	2.98
CRFR2α	N-terminal LBD 3–154	3N96 (Pal et al., 2010)	Urocortin-1 (25–40)	2.75
	N-terminal LBD 3–154	3N95 (Pal et al., 2010)	Urocortin-2 (23–38)	2.72
	N-terminal LBD 3–154	3N93 (Pal et al., 2010)	Urocortin-3 (23–38)	2.50
CRFR-2β	N-terminal LBD 15–133	1U34 (Grace et al., 2004)	N/A	N/A NMR
	N-terminal LBD 39–133	2JNC (Grace et al., 2007)	No ligand	N/A NMR
	N-terminal LBD 39–133	2JND (Grace et al., 2007)	Astressin	N/A NMR
**PARATHYROID HORMONE RECEPTORS**				
PTH1R	N-terminal LBD 29–187	3L2J (Pioszak et al., 2010)	N/A	3.24
	N-terminal LBD 29–187	3C4M (Pioszak and Xu, 2008)	PTH(15–34)	1.95
	N-terminal LBD 29–187	3H3G (Pioszak et al., 2009)	PTHrP(12–34)	1.94
**VASOACTIVE INTESTINAL POLYPEPTIDE RECEPTORS**				
VIP2R	N-terminal LBD 26–118	2X57	N/A	2.10
**Others**				
PACAP receptor	N-terminal LBD 22–143	2JOD (Sun et al., 2007)	PACAP (6–38)	N/A NMR
	N-terminal LBD	3N94 (Kumar et al., 2011)	PACAP (6–38)	1.80
GHRHR	N-terminal LBD 34–123	2XDG	N/A	1.95

In this review, we will focus on how the extracellular, transmembrane, and cytoplasmic domains of family B GPCRs (Figure 3) carry out the functions of peptide hormone recognition and signal transduction across the cell membrane. We will also discuss how this signaling process is modulated by accessory proteins (Figure 3). On the basis of the two-domain model (Figure 3), we will first discuss the recognition and binding of peptide ligands in the extracellular domains, including the N-terminal and juxtamembrane domains (Section Extracellular Domain: Ligand Binding). Then, we will discuss ligand interactions and ligand-induced helical movements in the transmembrane domain (Section Transmembrane Domain: Ligand-induced Helical Movements). Subsequently, we will review how activated family B GPCRs couple to G proteins in cytoplasmic domain (Section Cytoplasmic Domain: G Protein Coupling). We will also provide a brief overview of accessory proteins that regulate the activity of family B GPCRs (Section Interaction with Accessory Proteins). Finally, we will conclude with a summary and outlook of mechanistic studies of family B GPCRs (Section Challenges and Outlook). Through these discussions, we hope to provide a systematic review of the current understanding of molecular activation mechanism of family B GPCRs.

## Extracellular domain: ligand binding

The extracellular domain of family B GPCRs contains two parts: the N-terminal domain and the juxtamembrane domain (Figure 3). The latter is composed of three extracellular loops and the peptide fragment at the junction between the N-terminal domain and helix 1. The N-terminal and juxtamembrane domains work together to sense the peptide hormones in the initial step of signal transduction. Solved structures of the extracellular domains bound to their cognate peptide ligands (Table 2) provide crucial information about mechanisms of ligand recognition and selectivity. In this section, we will first discuss the two-domain binding model and then the specific molecular interactions of the ligands with the N-terminal and juxtamembrane domains of the receptors.

### Experimental evidence for the two-domain binding model

The two-domain binding model, as described in Figure 2, has been widely used to describe the binding of peptide hormones to family B GPCRs (as reviewed in Pal et al., 2012), which is supported by kinetic and reciprocal chimera studies.

In the kinetic studies, Förster Resonance Energy Transfer (FRET) experiments of parathyroid hormone (PTH) binding to parathyroid hormone receptor 1 (PTH1R) showed the first high affinity binding step occurs quickly (~0.10 s) and the second activation step slowly (~1 s) (Vilardaga et al., 2011). Although similar kinetic data from other members of the family is currently unknown, the two-step kinetics of receptor binding and activation may potentially be conserved in other family B GPCRs.

**Figure 4:**
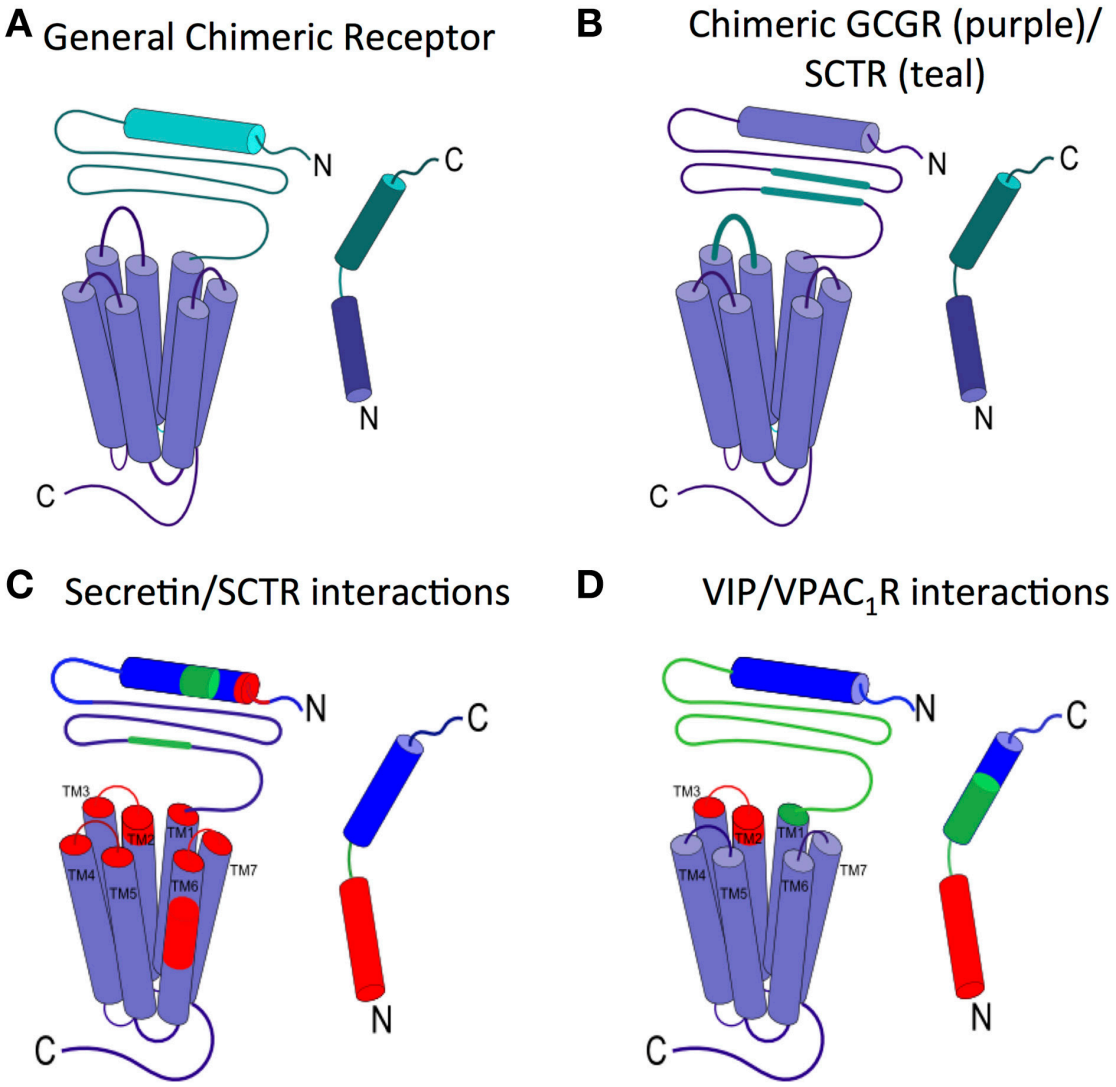
**Ligand interactions with the juxtamembrane domain**. **(A)** General chimeric receptor made from components of two receptors: one receptor's extracellular domain and C-terminus of the ligand (teal) and the other receptor's transmembrane domain and N-terminus of the ligand (purple). **(B)** Binding studies of a chimeric GCGR (purple) with regions of SCTR (teal) implicate extracellular loop 1 and the middle region of the N-terminal domain in high affinity ligand binding (Bergwitz et al., 1996; Unson et al., 2002). **(C)** Photoaffinity cross-linking studies with Bpa show interactions between colored regions of secretin and the corresponding colored portion of SCTR. the long N-terminal fragment of secretin interacts deep in the transmembrane region of SCTR (Dong et al., 2002, 2004, 2008, 2011b, 2012). **(D)** Interactions between VIP and VPAC_1_R as determined by mutations and photoaffinity cross-linking studies show similar interaction regions as secretin/SCTR (Couvineau et al., 1995; Du et al., 1997; Tan et al., 2003, 2006; Ceraudo et al., 2008).

The two-domain model is also supported by two representative chimera studies, in which one GPCR is created from pieces of two different receptors and one ligand from pieces of the two corresponding cognate peptide ligands (Figure 4A). In the first study, Bergwitz et al. designed a chimeric system using calcitonin receptor (CTR) and PTH1R, and determined the activation of the chimeric receptors by quantifying cAMP accumulation after treatment with two chimeric peptide hormones (Bergwitz et al., 1996). The chimeric peptide hormones with calcitonin at the C-terminus most strongly activated the chimeric receptor with CTR's N-terminal domain. Similarly, the peptide ligand with the PTH C-terminus most strongly activated the chimeric receptor with the PTH1R's N-terminal domain (Bergwitz et al., 1996). In the second study, Unson et al. mutated regions of the N-terminal domain and juxtamembrane domain of the glucagon receptor (GCGR) to the corresponding regions of the secretin receptor (SCTR, Figure 4B; Unson et al., 2002). Chimeric receptors containing secretin residues for the N-terminal domain residues 126–137 or the extracellular loop 1 residues 206–219 showed the largest decrease in both ligand binding and receptor activation upon binding to the glucagon peptide (Figure 4B). Interestingly, a chimeric GCGR/SCTR with SCTR residues in extracellular loop 1 residues 220–231 showed no change in binding affinity to the glucagon peptide, but a decrease in receptor activation (Unson et al., 2002), further elucidating a specific region of the juxtamembrane domain involved in the second binding step and receptor activation, supporting the two-domain model.

### Ligand binding interactions with the N-terminal domain

**Figure 5:**
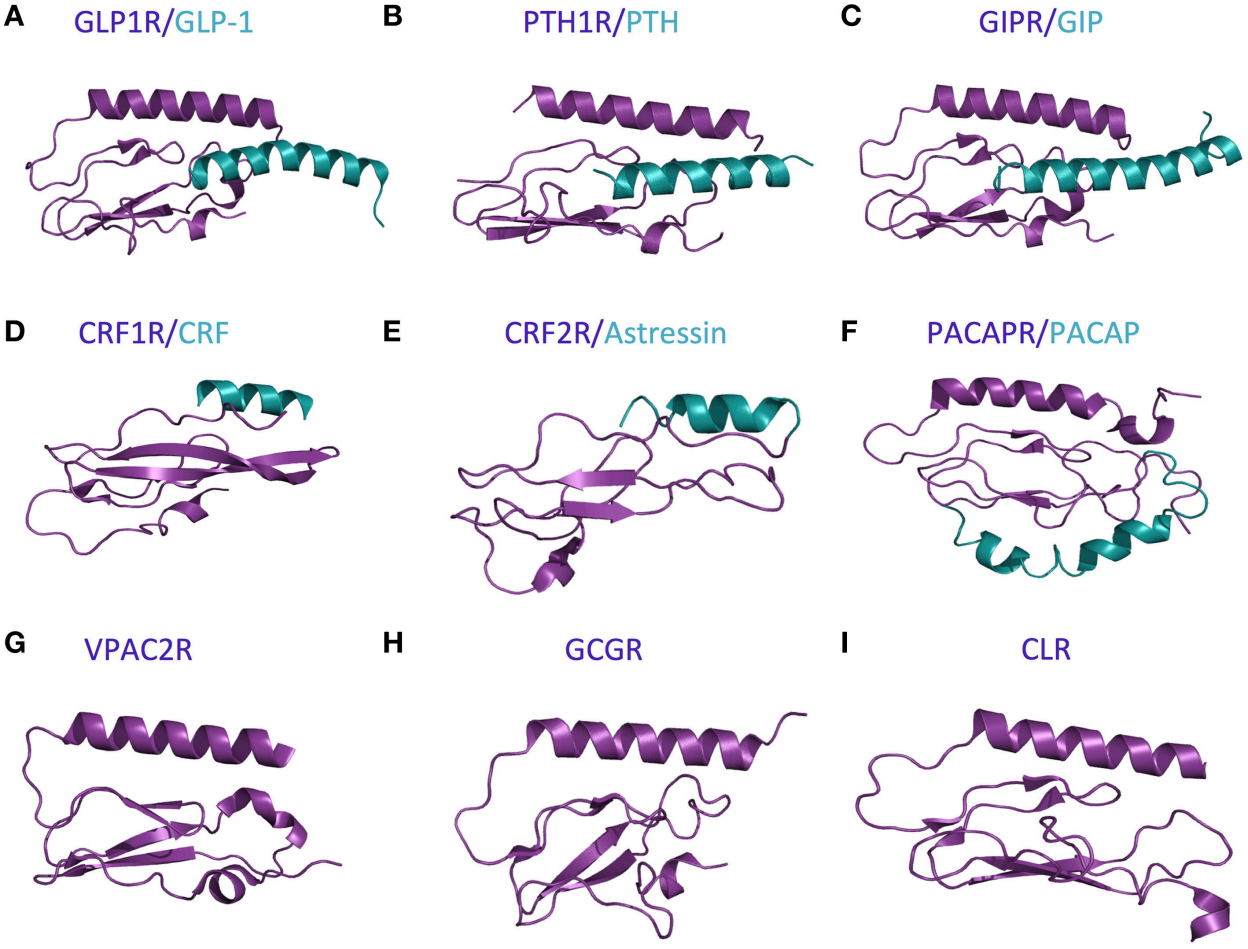
**Available crystal structures of the N-terminal domains of nine family B GPCRs**. **(A)** GLP-1 bound to GLP1R (Underwood et al., 2010). **(B)** PTH bound to PTH1R (Pioszak and Xu, 2008). **(C)** GIP bound to GIPR (Parthier et al., 2007). **(D)** CRF bound to CRF1R (Pioszak et al., 2008). **(E)** Astressin bound to CRF2R (Grace et al., 2004). **(F)** PACAP bound to PACAPR (Sun et al., 2007). **(G)** VPAC2R (PDB ID 2X57). **(H)** GCGR (Koth et al., 2012). **(I)** CLR (ter Haar et al., 2010).

**Figure 6:**
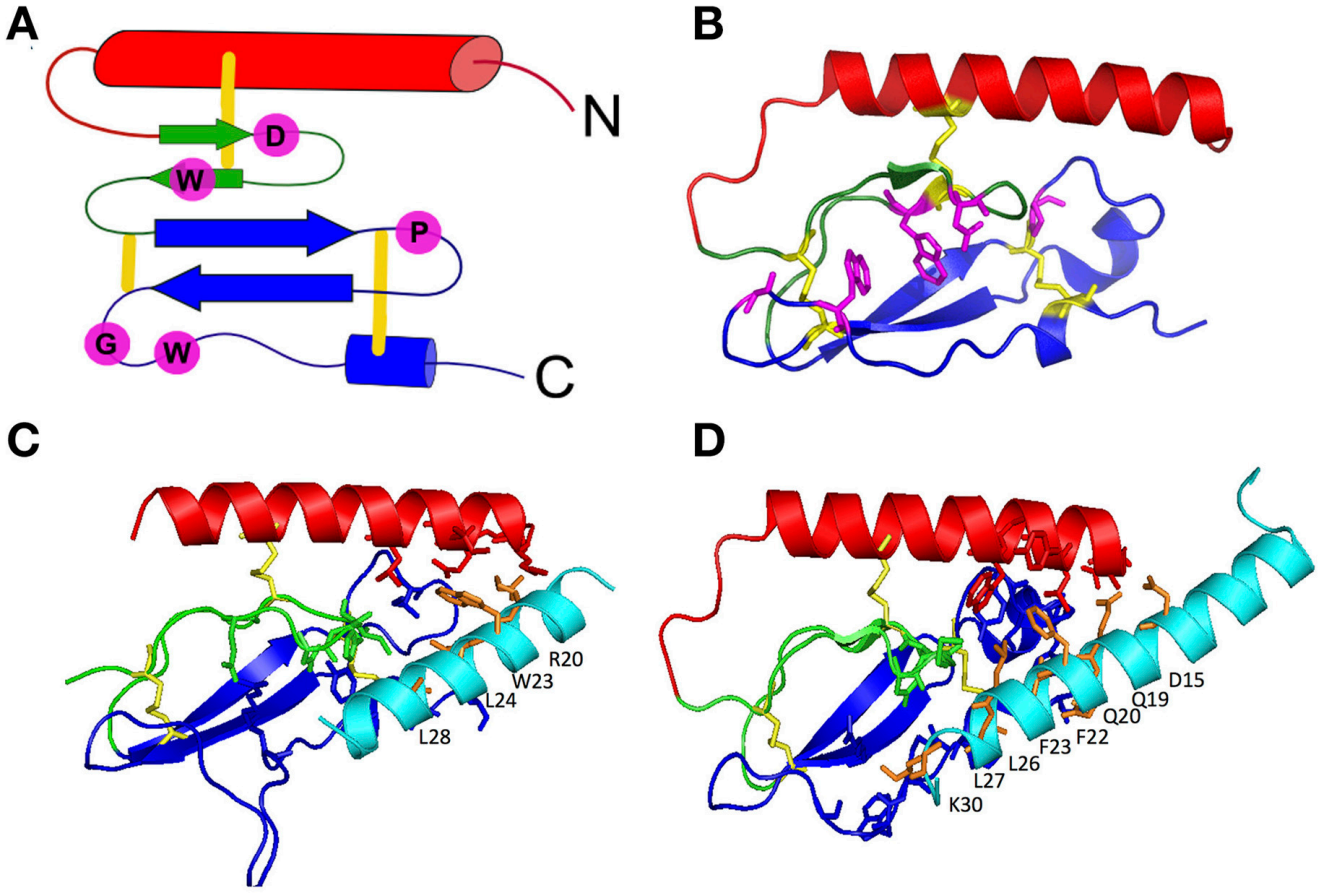
**The three-layered α-β-βα fold for the N-terminal domain of family B GPCRs**. **(A)** Schematic of the α-β-βα fold with conserved residues (pink) and disulfide bonds (yellow). **(B)** A representative crystal structure showing the α-β-βα fold, disulfide bonds and conserved residues (PDB 2QKH Parthier et al., 2007). **(C,D)** Important residues in peptide ligands for binding to PTH1R **(C)** and GIPR **(D)** identified by crystal contacts and mutagenesis studies. Important residues contacts are shown as sticks. Color codes: the top (red), middle (green), and bottom (blue) layers, three conserved disulfide bonds (yellow), conserved residues stabilizing the three-layer fold (pink), ligand residues important in binding affinity (orange; Pioszak and Xu, 2008).

The first step in the two-domain model—ligand binding to the N-terminus of receptors—is strongly supported by crystal structures of nine different ligands bound to the N-terminal domain of their respective receptors (Table 2 and Figure 5; Grace et al., 2004; Parthier et al., 2007; Sun et al., 2007; Pioszak and Xu, 2008; Pioszak et al., 2008, 2009; ter Haar et al., 2010; Underwood et al., 2010; Koth et al., 2012). These crystal structures reveal that the N-terminal domains contain a conserved three-layered α-β-βα fold (Figure 6) with the helical segment of the peptide hormone primarily interacting in the middle layer as if a “hotdog in a bun” (Figures 5A–C, 6) (Parthier et al., 2009; Pal et al., 2012). Six conserved cysteine residues form three interlayer disulfide bonds that stabilize the α-β-β α fold (Figure 6). Additional hydrophobic packing interactions and hydrogen bonds stabilize the second and third layers (as reviewed in Hollenstein et al., 2014). Sequence conservation indicates the importance of these stabilizing interactions, which likely involve five mostly conserved residues: Asp113, Trp118, Pro132 and Gly152, and Trp154 (PTH1R residues, pink in Figures 6A,B; Pioszak and Xu, 2008). These conserved residues and the structural similarities of the three-layered fold form a structural foundation for the two-domain binding model.

However, there are a few differences between the structures of the various receptor N-terminal domains, which likely provide ligand specificity. For example, Grace et al. determined the structure of the N-terminal domain of CRF2R (Figure 5E) using NMR and found the absence of the N-terminal α-helix that is conserved in the majority of the other crystal structures (Figure 5; Grace et al., 2004). More recent crystal structures of the N-terminal domains of CRF1R show the N-terminal α-helix tends to be shorter than the same region in other family B receptors (Figure 5D compared to Figures 5A–C,F–I; Pioszak et al., 2008). These structural characteristics cause the peptide hormone to interact with the N-terminal domain at a slightly shifted location compared to the other receptors including GLP-1R, PTH1R, and GIPR (Figures 5D,F compared to Figures 5A–C). Sequence alignment of the 15 Family B GPCRs supports the difference in the structure of the N-terminal α-helix as it shows a limited number of conserved residues in addition to the six conserved cysteine residues in the N-terminal domain (Pioszak et al., 2009).

Aside from structural data, biochemical studies further support the two-domain binding model (summarized in Supplementary Table S1). The reciprocal chimera studies that support the “hotdog in a bun” model also implicate the middle layer of the N-terminal domain in peptide hormone binding (Bergwitz et al., 1996). In addition, results from alanine scanning studies further determined the N-terminal domain residues necessary for ligand binding (Barwell et al., 2010). These alanine-scanning studies test receptor constructs with mutations in specific regions of a receptor for ligand binding affinity using radiolabeled ligands and for receptor activation using cell based cAMP accumulation assays. Depending on the location of the mutations, they might affect only the ligand binding affinity (the first binding event), only the receptor activation (the second binding event), or both responses. For example, mutations in the N-terminal α-helix (red in Figure 6A) of the calcitonin gene related peptide (CGRP) receptor show that mutating Leu32 and Leu34 to alanine decreases binding affinity (the first binding event) without decreasing cAMP production (the second binding event; Banerjee et al., 2006).

**Figure 7:**
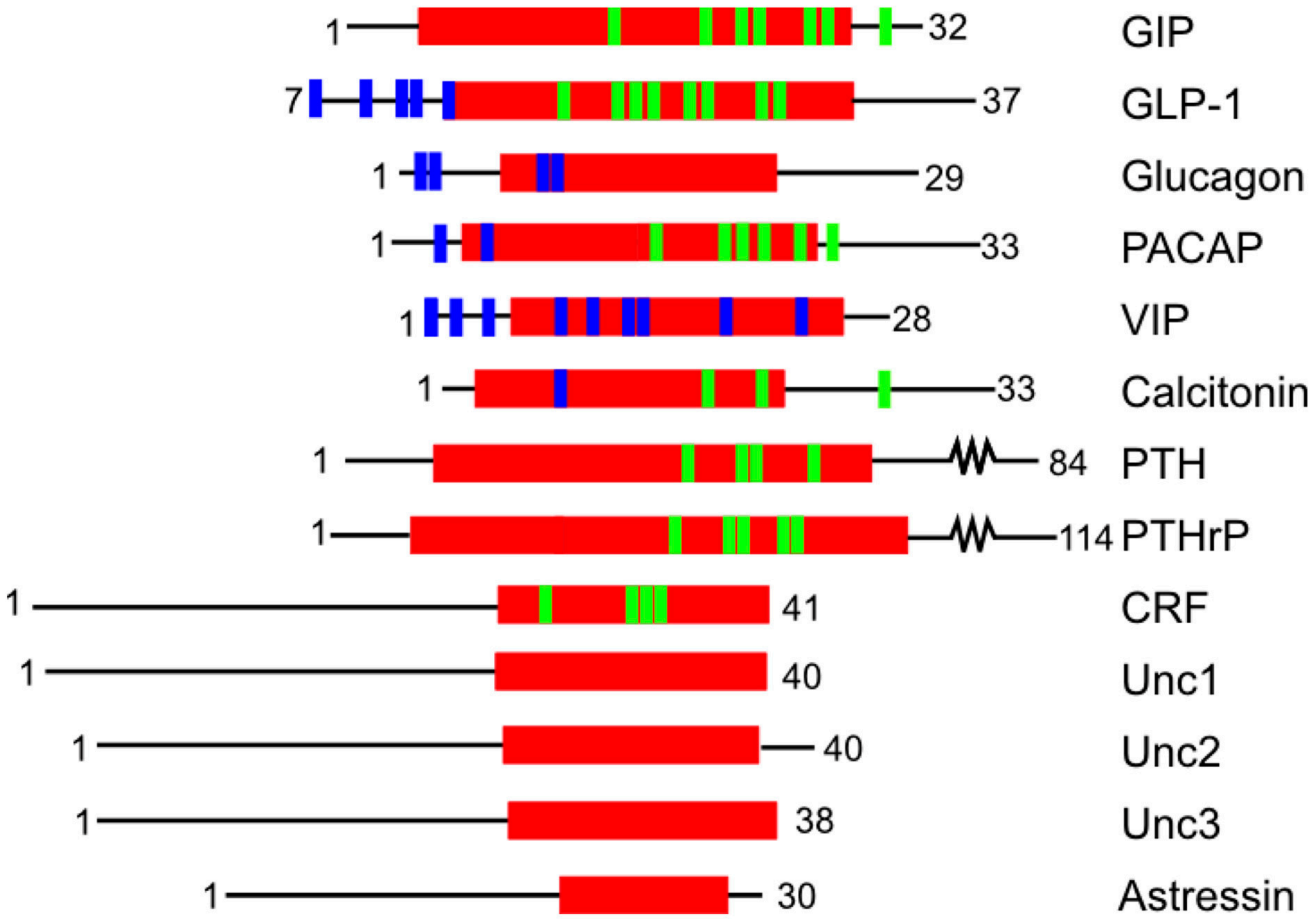
**Structure based alignment of family B GPCR ligands from the center of the α-helix (red)**. The blue and green lines show residues that decrease ligand-binding affinity when mutated. Blue lines indicate mutations that decrease ligand binding without specifying the region of the receptor where the interaction occurs. Green lines indicate residues implicated in ligand binding that interact with the N-terminal domain as confirmed by crystal structures. The data for the calcitonin receptor comes from cross-linking experiments (Adelhorst et al., 1994; Igarashi et al., 2002b; Perret et al., 2002; Dong et al., 2004; Pham et al., 2004, 2005; Parthier et al., 2007; Sun et al., 2007; Pioszak and Xu, 2008; Pioszak et al., 2008, 2009; Bourgault et al., 2009).

A complementary approach to uncovering the ligand binding determinants involves alanine scanning of the peptide hormone residues (Adelhorst et al., 1994; Nicole et al., 2000; Igarashi et al., 2002a,b; Bourgault et al., 2009; Dong et al., 2011b, 2013; Watkins et al., 2013). Figure 6 shows important results from alanine scanning studies of PTH and gastric inhibitory polypeptide (GIP). The residues labeled in orange in the peptide hormones significantly decrease binding affinity when mutated to alanine, confirming their importance in ligand binding (Parthier et al., 2007; Pioszak and Xu, 2008). In addition, one recent study of the secretin peptide with ligand binding and activation assays compared results from five receptors and found many N-terminal residues of the hormones are important for both ligand binding and biological activity, while C terminal residues tend to be important for binding affinity (Figure 7; Dong et al., 2011b). In combination with the crystal structures and N-terminal domain mutations, this provides a more complete view of the high affinity binding of the C-terminal region of the peptide to the N-terminal domain, the first step of the two-domain binding model.

### Ligand binding to the juxtamembrane domain

The second step of the two-domain binding model involves interactions between the N-terminus of the peptide and the juxtamembrane domain of the receptor, which has been less studied mainly due to the lack of structural information of full-length family B GPCRs. Photoaffinity cross-linking has been commonly used to probe these interactions and elucidate specific contacts between the peptide ligand and the receptor. In the photoaffinity studies, *p*-benzoyl-L-phenylalanine (Bpa) is incorporated in the hormone ligands as a probe. Upon exposure to UV light, the Bpa residue is cross-linked with the residues in the receptor within 9 Å. Hence, the site of interaction can then be determined by proteolytic Edman degradation followed by autoradiography (Miller et al., 2011). Photoaffinity studies on GLP-1R, SCTR, and PTH1R have identified important receptor/ligand interactions (Figure 4; Zhou et al., 1997; Chen et al., 2009; Dong et al., 2011a; Miller et al., 2011). For example, Dong et al. investigated secretin/SCTR interactions, with five different Bpa label positions and found the labels at the C-terminal end of the peptide (residues 15, 20, 24, 25) interacted with the N-terminal domain (blue, Figure 4C), while labels at the N-terminus of the peptide interact with extracellular loop 1 and regions of the transmembrane domain (red, Figure 4C; Dong et al., 2011a). The interactions identified support the second step of the N-terminal region of the ligand interacting with the juxtamembrane domain in the two-domain binding model.

Moreover, mutagenesis studies of the extracellular loops of receptors also show the importance of the juxtamembrane domain in ligand binding and receptor activation. For instance, Gkountelias et al. studied CRF1R. They mutated residues in all three of the extracellular loops and found that two mutations in extracellular loop 2 (Phe260 and Trp259) decreased both ligand binding affinity and receptor activation (Gkountelias et al., 2009). In addition, an alanine scanning study of the calcitonin receptor implicated extracellular loop 1 in ligand binding (Barwell et al., 2011). These results suggest that the juxtamembrane domain is involved in not only ligand binding but also receptor activation in the two-domain binding model.

A final important factor in ligand binding to the juxtamembrane domain is the structure of the N-terminal region of the peptide hormone. By analyzing the sequence of the N-terminal region of the peptide ligands, Neumann et al. identified conserved helix-capping motifs, which provide additional hydrogen bonds for stabilizing the configuration of the first four amino acids in the α-helices (Neumann et al., 2008). Based on the analysis, they proposed that this helix-capping can play an important role in receptor specificity and formation of α-helix in the peptide ligands upon binding to receptors (Parthier et al., 2009). These proposed functional roles of the helix-capping motifs may be very important and are worth further experimental validation.

Aside from specific interactions between the peptide hormone and the extracellular domain, other factors influence ligand binding affinity and specificity for a given receptor. One such situation occurs when a receptor binds more than one native ligand, with each ligand eliciting a distinct physiological response. For instance, PTH1R interacts with two native ligands, PTH and PTHrP. While both peptides initiate cAMP accumulation inside cells, continuous administration of individual ligands induces opposite physiological effects, with PTH increasing bone resorption and PTHrP stimulating bone formation (Mannstadt et al., 1999). Nonetheless, how the receptors distinguish between these different pathways remains unclear. In addition, allosteric effects from regions of the receptors not directly involved in ligand binding add complexity to the molecular mechanism of ligand recognition in family B GPCRs. For example in GLP-1R, a mutation across the membrane in intracellular loop 3 (ICL3) decreases the binding affinity of the GLP-1 ligand by 10-fold (Heller et al., 1996). Further investigation into allosteric effects is likely to uncover new mechanisms of ligand selectivity in family B GPCRs.

Both structural and biochemical results greatly increase the understanding of the interactions of the peptide hormone binding to the N-terminal and juxtamembrane domains, formulating the well accepted two-domain binding model. Although the process of peptide-hormone binding to the receptor is the most studied event of the signaling cascades, identifying interactions is only part of the story. Very little is known about how those interactions cause conformational changes that propagate the signal to the cytoplasmic side of the receptor, resulting in activation of G proteins. In the following section, we will summarize the current understanding of such conformational changes in the transmembrane region of family B GPCRs.

## Transmembrane domain: ligand-induced helical movements

The transmembrane domain (TMD) of family B GPCRs serves as the bridge for communication between extracellular ligand binding and the G-protein coupling at cytoplasmic regions. Ligand binding causes conformational rearrangements within the TMD, which triggers downstream signaling cascades via G protein activation. The structures of the ligand-bound N-terminal domain of many family B GPCRs have been reported. In 2013, TMD crystal structures of two family B GPCRs were published, (Hollenstein et al., 2013; Siu et al., 2013) a major step forward in the field. However, the structure of a full-length family B GPCR remains largely unknown. As a result, the mechanistic understanding of conformational rearrangements in the activation process of family B GPCRs had long been stagnant. In this section, we will analyze the TMD structures of two family B GPCRs in comparison to family A GPCRs. We will focus on the ligand interactions within the TMD regions, as well as the potential of designing small-molecule modulators for binding to the TMD region of family B GPCRs. Finally, we will evaluate possible conformational changes of the TMD upon activation in light of both the new structural information and prior biochemical studies.

### Structural comparison of CRF1R and GCGR and key residues involved

**Figure 8:**
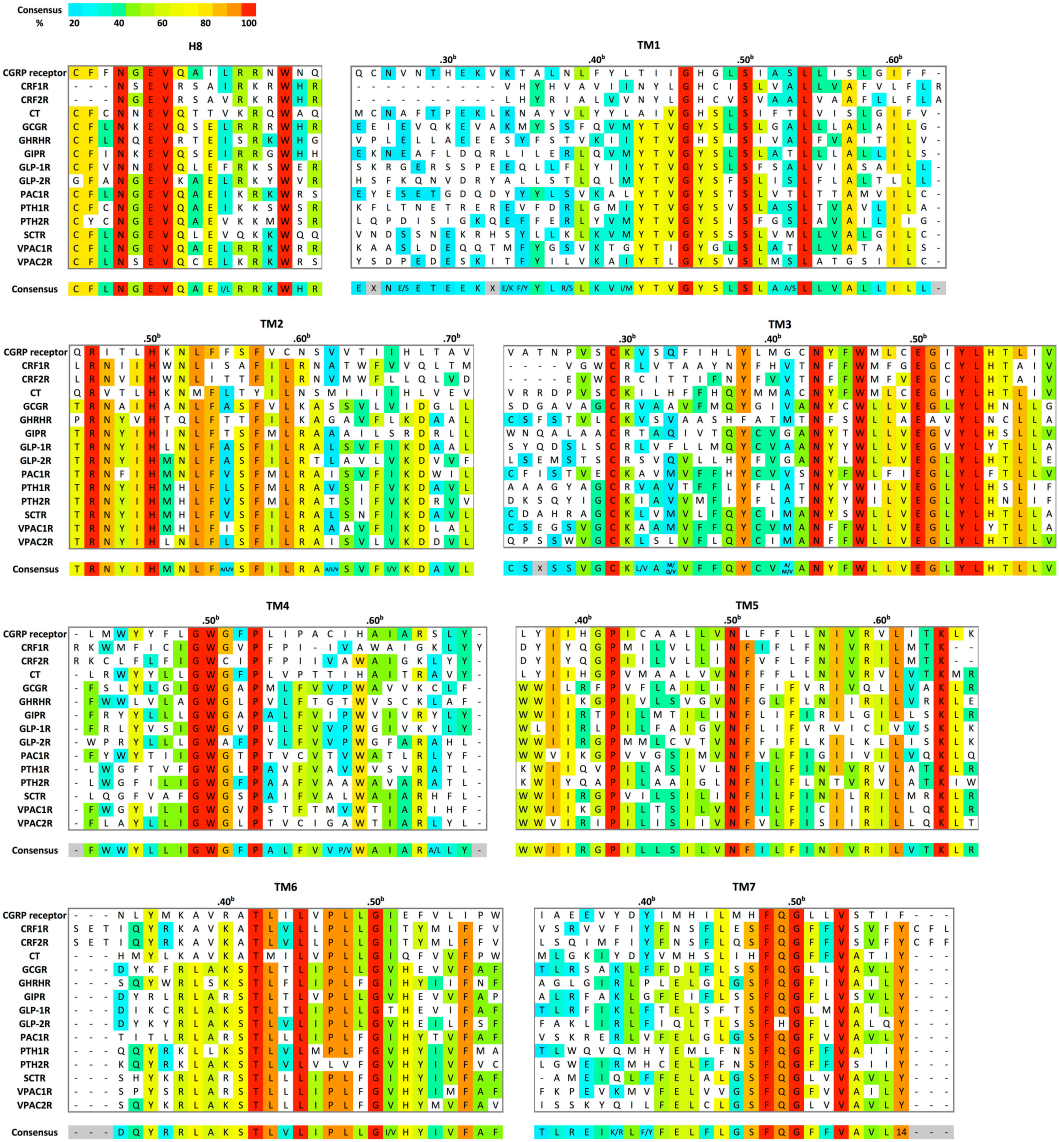
**Structural-based sequence alignment of the transmembrane helices and helix 8 of family B GPCRs**. For every position in the transmembrane region, the most conserved residue is color coded with its percent consensus (color coded from cyan 20% to red 100% consensus). The residues in transmembrane helices are numbered according to Wootten numbering scheme (Box 1 in Figure 10).

To date, crystal structures of the TMD of two family B GPCRs are available. They include human corticotropin-releasing factor receptor 1 (CRF1R) in complex with the small-molecule antagonist CP-376395 at 3.0 Å resolution, (Hollenstein et al., 2013) and human glucagon receptor (GCGR) in complex with the antagonist NNC0640 at 3.4 Å resolution crystalized with apocytochrome *b562*RIL from *E.coli* fused at the N-terminus (Siu et al., 2013). CRF1R and GCGR share about 30% sequence identity within the TMD (Figure 8). Here, we compare their structural characteristics with respect to family A GPCRs.

**Figure 9:**
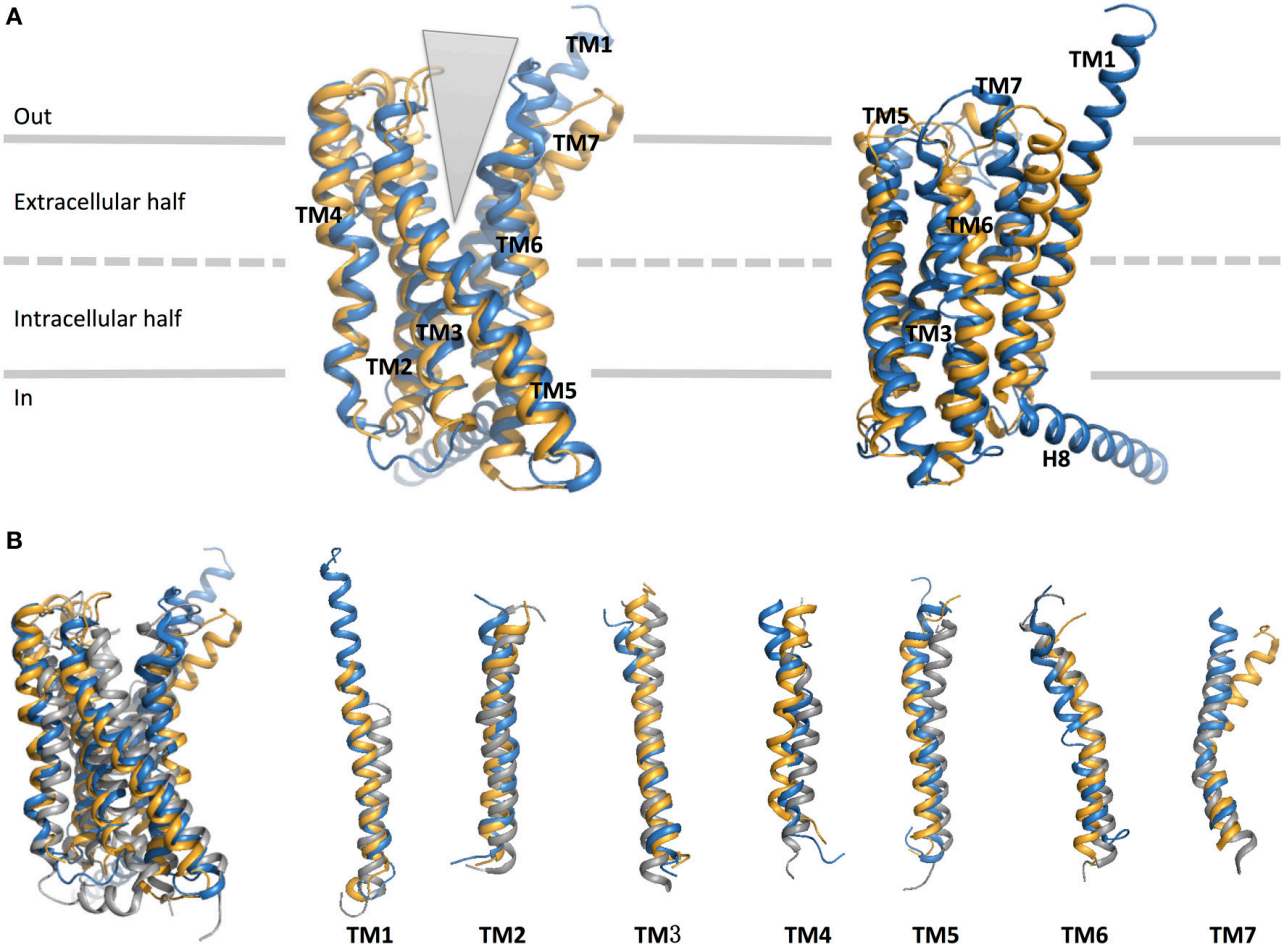
**Structural alignment of CRF_1_ (gold, PDB ID 4K5Y) and GPCR (blue, PDB ID 4L6R)**. **(A)** The receptors are viewed from two different angles from within the membrane. The TM helices are labeled and comprise the two halves of the V-shape open configuration. **(B)** Structural comparison of the two family B GPCRs with a family A GPCR, dopamine D_3_ receptor (gray, PDB ID 3PBL), in its inactive form. Individual TM helices are shown after superposition of the three receptors.

As expected, the hallmark 7TM helical bundles of family A GPCRs are observed in both CRF1R and GCGR. Figure 9 presents the superimposition of the crystal structures of the 7TM of the two family B GPCRs and a representative family A GPCR [dopamine D_3_ receptor (Chien et al., 2010)] in their inactive conformations. While the structural conservation in the two family B GPCRs appears highest among the cytoplasmic half of TM helices, the extracellular half shows more divergence. This suggests each receptor has a characteristic, highly selective ligand-binding site. Also, it is worth noting that GCGR has an extended, long TM1 as compared to family A GPCRs, which is seen in the sequence of most family B GPCRs (Figure 8). This helical fragment, extending into the extracellular and juxtamembrane domains, may play an important role in ligand binding and signal transduction of the transmembrane region. On the other hand, the structural similarity of the cytoplasmic half of the helices suggests conformational rearrangements associated with G protein binding upon activation may be more conserved because a large number of GPCRs interact with the same, less diverse G proteins. The TMD structures of the two family B GPCRs show that the extracellular sides of the transmembrane domains assume a “V” shape that is more open toward the extracellular side than any of the family A GPCR structures (Figure 9). The “V” shape conformation presents a large, solvent-filled cavity accessible to extracellular ligand binding. One side of the “V” shape is formed by TM1, TM6, and TM7, and the other side is by TM2–TM5 (Figure 9A). These bundles of helices are similar to those observed in rhodopsin and other family A receptors (Crocker et al., 2006).

**Figure 10:**
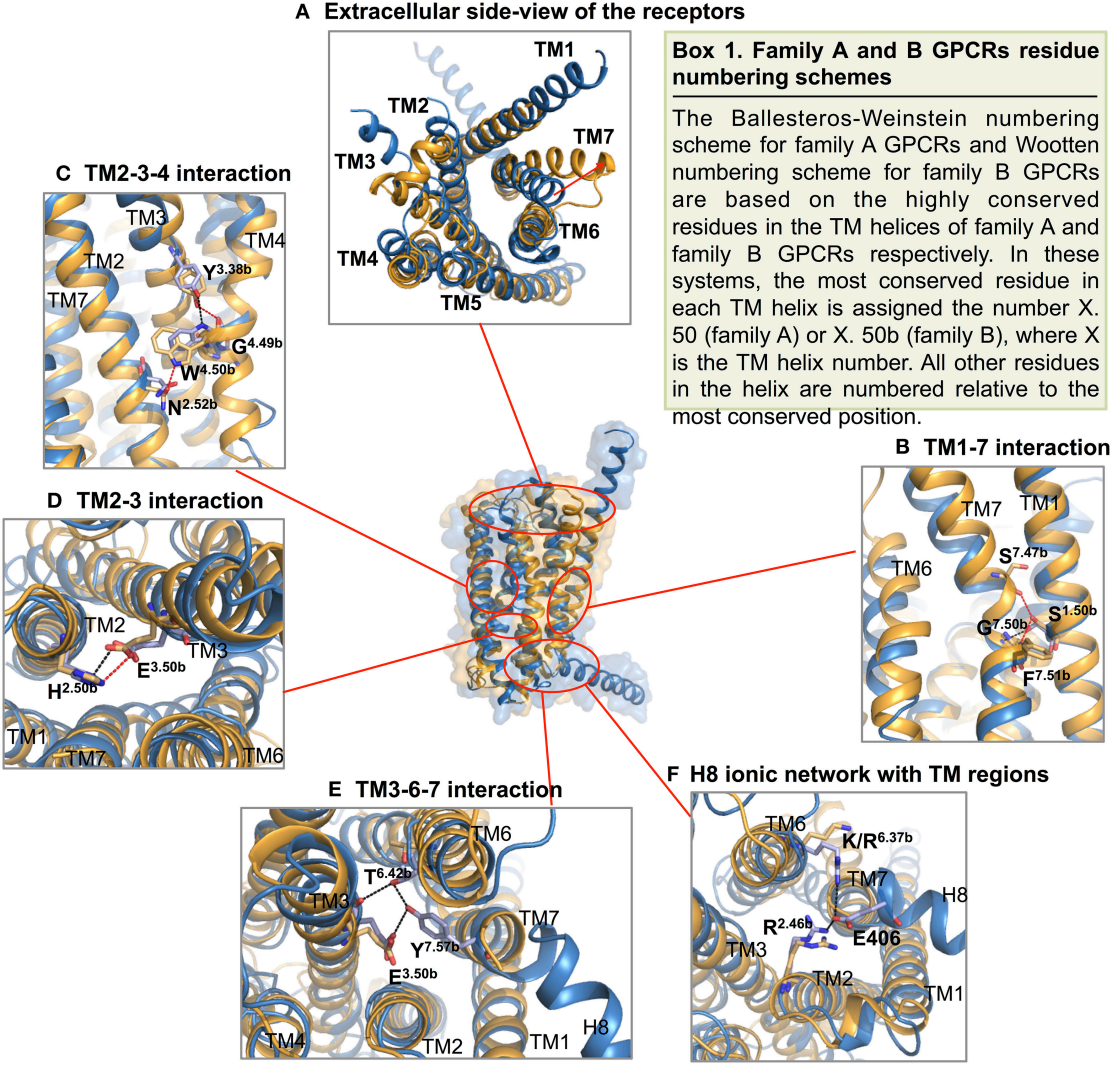
**Structural features of family B GPCRs**. Conserved structural features in CRF1R (yellow) and GCGR (blue) are highlighted within the superposition of the two structures. **(A)** The receptors viewed from the extracellular side; **(B,C)** The conserved CRF1R and GCGR residues involved in TM1-7 and 2-3-4 interface interactions at the extracellular half of the V-shape open configuration; **(D,E)**. The conserved residues involved in TM2-3 and 3-6-7 interactions at the intracellular half of the V-shape configuration; **(F)** GCGR residues Glu 406 of H8 forms an ionic network interacting with TM2 and TM6. Polar contacts are indicated by red (CRF1R) or black (GCGR) dashes.

Turning to TM1, TM6, and TM7 on one side of the “V” shape, TM6 and TM7 of both receptors point away from the center of the helical bundle. In CRF1R, this region is even further away from the center of the helical bundle compared to GCGR. Similar interhelical interactions in CRF1R and GCGR contribute to the stabilization of the extracellular half of the transmembrane domain. In CRF1R, TM1 packs against and stabilizes a kink in TM7 at Gly356^7.50b∕7.46^ (Figure 10 Box 1) through formation of hydrogen bonds between highly conserved Ser130^1.50b∕1.46^ on TM1 and Phe357^7.51b∕7.47^ and Ser353^7.47b∕7.43^ on TM7 (Figure 10B). Although the same interaction is not clearly observed in the crystal structure of GCGR, the conserved residues Ser152^1.50b∕1.46^ on TM1 and Gly393^7.50b∕7.46^ on TM7 may perform a similar role, resulting in the kinked structure in TM7 that causes the extracellular halves of TM6 and TM7 to tilt away. The kink in TM7 is similar to the one revealed in the crystal structures of family A GPCRs situated at the highly conserved NPxxY motif. In addition, the TM6 and TM7 regions of both receptors exhibit high temperature factors, indicating structural flexibility and possible movements upon ligand binding and activation.

On the other side of the “V” shape, TM4 in both receptors contains the highly conserved family B GPCR sequence motif of GWGxP. Although the TM4 helices of the two receptors do not superimpose perfectly (Figure 9), this GWGxP motif plays an important structural role in stabilizing the configuration of TM2, TM3, and TM4 (Hollenstein et al., 2014). In CRF1R, TM4 bends at Gly235^4.49b∕4.49^ and points Trp236^4.50b∕4.50^ toward TM2 and TM3 within the conserved motif. Hydrogen bonds are formed between Trp236^4.50b∕4.50^ and Asn157^2.52b∕2.45^ on TM2 and Tyr197^3.38b∕3.34^ on TM3 (Figure 10C). In GCGR, while residues Gly271^4.49b∕4.49^ and Trp272^4.50b∕4.50^ on TM4, Asn179^2.52b∕2.45^ on TM2 and Tyr233^3.38b∕3.34^ on TM3 are conserved, hydrogen bonds are formed differently between Trp272^4.50b∕4.50^ and Tyr233^3.38b∕3.34^ (Figure 10C). The inconsistency may result from the ambiguous electron density in the TM4 region of GCGR. Nonetheless, given the high resolution across this region in CRF1R, the observed interactions associated with the GWGxP motif in CRF1R may exist in all family B GPCRs (Hollenstein et al., 2013, 2014).

Moving from the extracellular side to the cytoplasmic side of the transmembrane domains, His^2.50b∕2.43^ on TM2 and Glu^3.50b∕3.46^ on TM3 are in close proximity in both structures, and their interaction is proposed to be functionally and structurally important (Schipani et al., 1995, 1997; Heller et al., 1996; Hjorth et al., 1998; Vohra et al., 2013; Wootten et al., 2013). Toward the end of TM7, Tyr400^7.57b^ of GCGR forms hydrogen bonds with residues Glu245^3.50b^ on TM3 and Thr351^6.42b^ on TM6 and is positioned in a similar conformation to family A GPCRs that is associated with activation (Rasmussen et al., 2011). Interactions at the equivalent Tyr363^7.57b^ of CRF1R were not clearly present in the crystal structure, possibly because helix 8 was removed in the construct, which may have affected the orientation and length of TM7. In fact, this tyrosine residue is conserved among family B GPCRs (Figure 8), and is predicted to be associated with the activation and interaction with the G protein (Vohra et al., 2013; Wootten et al., 2013), as discussed later in Section Activation and Allosteric Transition.

Two additional structural features that may be functionally important in the GCGR structure include the additional N-terminal helical turns of TM1 extending far into the extracellular space and an unusually long helix 8 connected to TM7 (Figure 9). Even though these structural features may result from artifacts during crystallization, the extension of TM1 may be important in restricting the position of the N-terminal domain for ligand binding. In addition, the unusually long helix 8 connecting to TM7 bends slightly toward the cytoplasm and has its N-terminal involved in interactions with TM 3 and TM6 (Figure 10F), which may have functional significance.

### Ligand recognition by the transmembrane domain and druggability of family B GPCRs

Although there is no clear universal cognate peptide ligand-binding site within TMD of family B GPCRs, its location is suggested to be associated with the TMD (Coopman et al., 2011; Miller et al., 2011). The crystal structures of CRF1R and GCGR reveal ligand binding interactions surprisingly deep into the TMDs with wide binding pockets resulting from the large distances between extracellular side of TM2 and TM7, and between that of TM3 and TM7. The deep and wide binding pockets were not observed in any family A GPCRs.

**Figure 11:**
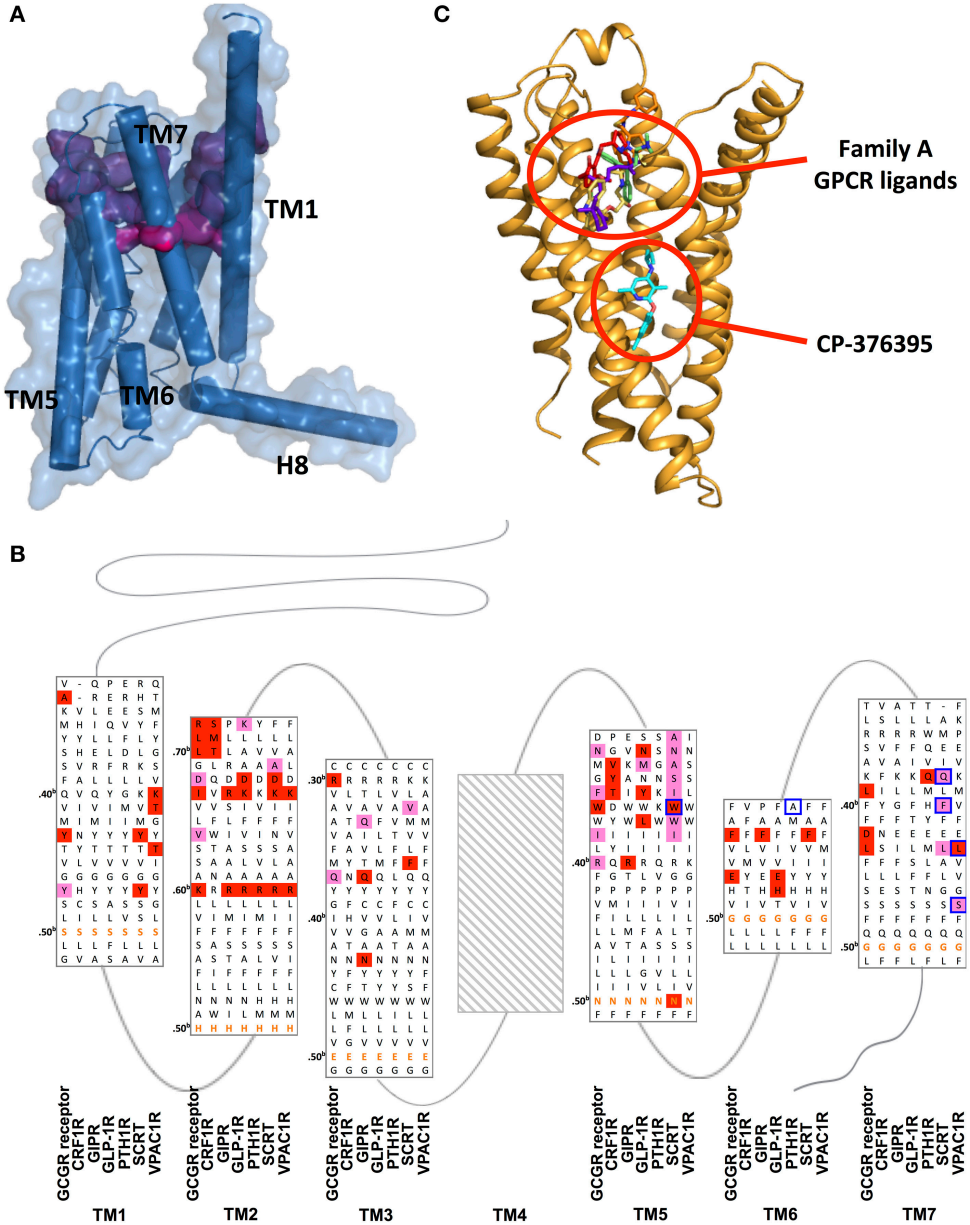
**Ligand binding sites of family B GPCRs**. **(A)** Peptide binding pocket of GCGR deep into the TMD. The surface of peptide-binding residues is color-coded regarding the depth (from purple-shallow to magenta-deep). **(B)** Mutation studies on effects of peptide binding in GCGR, CRF1R, GIP, GLP-1R, PTH1R, SCTR, and VPAC_1_R are mapped on a structure-based sequence alignment. Colored residues show 4–10-fold (pink), and >10-fold (red) effects on K_i_/IC_50_ values for peptide or ligand potency/EC_50_ value. The most conserved residues in TM1–7 of family B GPCRs (X.50^b^, Figure 10 Box 1) are bolded in orange. Receptor residues that covalently bind peptide ligands in photo-crosslinking or cysteine-trapping studies are boxed blue. Note that TM regions are not displayed in full length. No mutagenesis data for residues of TM4 was found and Supplementary Table S1 contains experimental details. **(C)** The location of CP-376395 in the CRF_1_R structure is compared to that of selected family A receptor ligands.

The highly conserved N-terminal motif of family B peptide ligands, which precedes the N-capping motif essential in receptor activation, has been proposed to reach through the juxtamembrane domain deep into the TMD. Upon interaction, the helical segment of the extra long TM1 in the juxtamembrane region is suggested to take a flexible helical structure and thereby position the N-terminal domain relative to the TMD in favor of ligand binding. Moreover, the N-capping of the peptide is suggested to stabilize the receptor-peptide complex (Neumann et al., 2008; Parthier et al., 2009), which is the second step of the two-domain binding model, as discussed above in Section Extracellular Domain: Ligand Binding. Early mutagenesis and photoaffinity studies on family B GPCRs including PTH1R, GLP-1R, and SCTR as well as recent structural studies of CRF1R and GCGR all provide evidence supporting the deep insertion (Figures 11A,C; Supplementary Table S1; Liaw et al., 1997; Mathi et al., 1997; Tseng and Lin, 1997; Di Paolo et al., 1998, 1999; Hjorth et al., 1998; Cascieri et al., 1999; Xiao et al., 2000; Gardella and Jüppner, 2001; Solano et al., 2001; Perret et al., 2002; Unson et al., 2002; Gensure et al., 2003; Runge et al., 2003; Dong et al., 2004, 2012; Grace et al., 2004; Hoare et al., 2006; Assil-Kishawi et al., 2008; Ceraudo et al., 2008, 2012; Prévost et al., 2010; Underwood et al., 2010; Yaqub et al., 2010; Coopman et al., 2011; Miller et al., 2011; Roberts et al., 2011; Donnelly, 2012; Koole et al., 2012; Koth et al., 2012; Wootten et al., 2013). For instance, Wright and Rodbell showed that the first six residues of the glucagon peptide interact with TMD of GCGR (Wright and Rodbell, 1979). Moreover, mutagenesis studies of GCGR also showed that mutations deep in the TMD directly affect glucagon binding, which involved residues Tyr149^1.4b^, K187^2.60b^, Val191^2.64b^, Gln232^3.37b^, Glu362^6.53b^, and Leu386^7.43b^ (Figure 11; Cascieri et al., 1999; Perret et al., 2002; Unson et al., 2002; Runge et al., 2003; Prévost et al., 2010; Roberts et al., 2011; Siu et al., 2013). A full receptor-ligand model of the glucagon bound GCGR generated by a combination of structural information of N-terminal domain-ligand complexes and the recent GCGR transmembrane crystal structure also showed a similar binding mode (Hollenstein et al., 2014). Moreover, the results of a series of mutagenesis studies summarized in Figure 11B provide additional support for the deep binding site in the TMDs, showing that homologous residues in family B peptide ligands interact with regions deep into the TMDs.

The relatively large N-terminal regions of family B peptide ligands may be useful in explaining the wider binding pocket. In fact, the N-terminus of the CRF peptide that binds CRF1R is significantly longer than the N-terminus of the glucagon peptide that binds GCGR (Parthier et al., 2009; Hollenstein et al., 2014). This may explain the wider pocket in CRF1R around the extracellular end of TM6, TM7, and extracellular loop 3, suggesting that the receptors can accommodate large peptide ligands by positioning the TM helices to form a larger binding pocket (Hollenstein et al., 2013).

The deep and wide binding pockets revealed by the two structures also provide new insights into designing molecules to modulate family B GPCRs. It is worth noting that the crystal structure of CRF1R in complex with CP-376395 uncovered an unusual, small-molecule binding pocket that is deep in the intracellular half of the receptor TMD, over 15 Å away from the any ligand-binding site in the known family A GPCR structures (Figures 11A,C; Okada et al., 2004; Cherezov et al., 2007; Chien et al., 2010; Wu et al., 2010; Shimamura et al., 2011; Hollenstein et al., 2013). The CP-376395 binding site is defined by TM3, TM5, and TM6 and features both hydrophobic and hydrophilic environments that are compatible with drug-like small organic molecules. It is proposed that CP-376395 acts as an antagonist by stabilizing the inactive conformation of CRF1R, preventing TM6 from moving away from the center of the TM bundle toward the membrane, a widely accepted activation mechanism for family A GPCRs for G protein docking (Bortolato et al., 2014). The residues in direct contact with CP-376395 in fact show high sequence identity among family B GPCR (Figures 11B,C), thus this region can potentially be exploited to design small-molecule drugs (Cascieri et al., 1999).

### Activation and allosteric transition

The activation mechanism and transmembrane movements of family A GPCRs have been extensively studied; however, very little has been explored in family B GPCRs. As elaborated by structural and biophysical studies of rhodopsin and other family A GPCRs (Tehan et al., 2014), the most profound allosteric transitions include a large outward movement of TM6 (Ballesteros et al., 2001) and the disruption of the ionic lock between the E/DRY motif on TM3 and acidic residues on TM6 upon activation (Palczewski et al., 2000; Shapiro et al., 2002; Kobilka and Deupi, 2007; Yao et al., 2009). In addition, TM7 bends inward and repositions the tyrosine residue in the conserved NPxxY motif, which connects to helix 8 to prevent the reverse movement of TM7 (Prioleau et al., 2002; Fritze et al., 2003). The bending stabilizes the receptor in an open state. In this state, TM5 repacks against TM6, opening the interaction surface for the G protein. These helical movements and switches provide additional platforms to interact with G proteins. The outward movement of TM6 creates an interacting pocket for the C-terminus of G_α_. Additional interaction sites of G_α_ lie in TM3, TM5, and intracellular loop 2 (ICL2) of the receptor (Janz and Farrens, 2004; Kobilka and Deupi, 2007; Choe et al., 2011). Nonetheless, these proposed helical movements found in family A GPCRs have remained almost unexamined in family B GPCRs.

Instead of structural and biophysical characterizations, most studies of transmembrane conformational changes in family B GPCRs have focused on mutagenesis to identify key residues and motifs involved in activation (Figure 11B). The structures of CRF1R and GCGR further confirmed the mutagenesis studies with a few highly conserved transmembrane motifs that are potentially important in the activation of family B GPCRs. First, while GCGR and CRF1R do not have the hallmark ionic lock between TM3 and TM6 in family A GPCRs, the H-bonding interaction observed between His^2.50b∕2.43^ on TM2 and Glu^3.50b∕3.46^ on TM3 may play an equivalent role, breaking upon activation, which allows outward movements of the helix bundle (Hollenstein et al., 2013, 2014; Siu et al., 2013). Second, the conserved NPxxY motif connecting TM7 to the cytoplasmic helix 8 of family A GPCRs is also missing in family B GPCRs. However, the tyrosine of that motif is still highly conserved across family B GPCRs (Figure 8). In fact, in GCGR, Tyr400^7.57b^ forms hydrogen bonds with residues on TM3 and TM6 and is positioned in a conformation similar to the conformation in family A GPCRs that is associated with activation. In CRF1R, alanine mutations at the equivalent position of Tyr363^7.57b^ shift the conformation of the receptor toward an inactive state. This indicates the importance of the conserved tyrosine residue in TM7 in family B GPCR activation, potentially stabilizing the receptor in an open and active conformation. These speculated functions of the conserved residues and motifs in family B GPCRs require careful experimental validation.

## Cytoplasmic domain: G protein coupling

**Figure 12:**
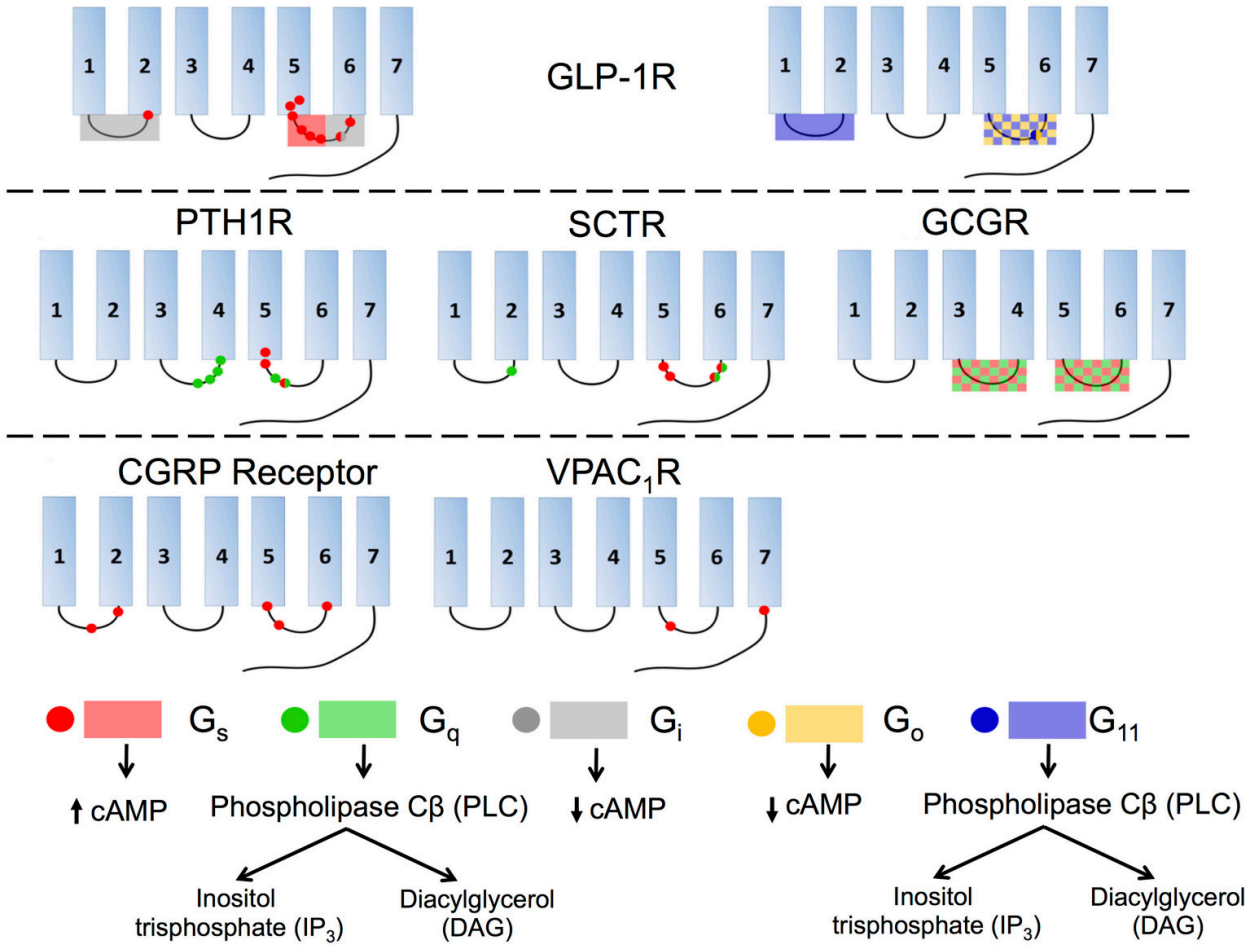
**Interactions of various isoforms of G proteins with the intracellular loops of six family B GPCRs and the downstream signaling pathways activated by the isoforms**. Red indicates G_s_, green G_q_, gray G_i_, yellow G_o_, and blue G_11_. Checkered boxes show two G proteins interact with the same loop. The red box on GLP-1R ICL3 (top left) shows the N-terminal half of the loop associates with G_s_ and the C-terminal part of the ICL3 associates with G_i_.

Conformational changes in the TMD, as discussed above, transduce signals from extracellular domains to intracellular domains, selectively activating various isoforms of G proteins that trigger specific downstream physiological responses (bottom, Figure 12; Ritter and Hall, 2009). In this section, we will review the general model for interaction of GPCRs and G proteins, emphasizing structural features important for the interaction in family B GPCRs. We will also discuss the proposed structural changes within the cytoplasmic regions upon activation of G proteins.

### General GPCR/G protein interaction model

Two models have been proposed for GPCR coupling to G proteins (Moreira, 2014). These models differ in how and when GPCRs interact with G proteins. The first one is the collision model, where GPCRs and G proteins diffuse freely within the cell membrane and interact via collision (Gilman, 1987). This model, however, is not sufficient to explain the rapid response of many GPCR-mediated pathways, where activation occurs within milliseconds (Vilardaga et al., 2003). The second model involves pre-assembled GPCR/G protein complexes that are formed before ligand binding to receptors. This model has been increasingly accepted and supported by a few experimental studies (Galés et al., 2005, 2006; Qin et al., 2011).

While the collision and pre-assembled models are mostly examined using family A GPCRs (Jastrzebska et al., 2010), they carry particular significance in understanding signaling processes of family B GPCRs. On one hand, previous studies show that the pre-coupling of different G proteins could switch the ligand selectivity in family B GPCRs and thereby modulates the receptor's functions (Ayoub and Pin, 2013). On the other hand, recent evidence suggests that binding of different native ligands with a single GPCR can selectively activate different G proteins. It is currently unknown if the ligand binding influences the selectivity of coupling to various G proteins in the collision model, or if the G protein coupling allows the receptor to change the binding affinity to different ligands in the pre-assembled model. Thus, examining these two models in family B GPCRs is important to unravel the intricacy of the signaling processes of family B GPCRs.

To explore the selectivity in ligands and G proteins for family B GPCRs, PTH1R has been commonly used as a model system (Ferrandon et al., 2009). PTH1R contains two native ligands, PTH and PTHrP. These two ligands share a high degree of structural and sequential similarities. However, recent FRET studies demonstrated the two ligands interact with PTH1R in distinct ways (Ferrandon et al., 2009). While both ligands bind with similar affinity to PTH1R when the receptor is pre-assembled with G_s_, PTHrP has a lower affinity when the receptor is not bound to G_s_ (Hoare et al., 2001; Dean et al., 2008; Okazaki et al., 2008). Whether this ligand biased signaling is characteristic of all family B GPCRs requires future studies.

In addition, whether GPCRs function as monomers or oligomers is important in G protein coupling, but still controversial. On one hand, GPCRs, except for the family C members, are proposed to be monomeric and couple to one G protein after receptor activation (as reviewed in Gurevich and Gurevich, 2008). On the other hand, recent evidence supports the idea that GPCRs may function in dimers or higher-order oligomers (Roed et al., 2012). The oligomeric state of the receptor might affect the receptor's ability to couple to and activate different G proteins, which would lead to activation of different downstream signaling pathways (Gao et al., 2009; Harikumar et al., 2012). Although a few studies have used co-immunoprecipitation, BRET, and FRET to understand GPCR oligomerization (Roed et al., 2012), the effect of the implicated oligomerization of family B GPCRs on G protein coupling requires further examination.

A detailed structural characterization of the GPCR/G-protein complex would, of course, provide accurate information about the interactions between the receptor and G protein. However, such structures are only available for two family A GPCRs, rhodopsin and β2 adrenergic receptor (β2AR) (Scheerer et al., 2008; Choe et al., 2011; Rasmussen et al., 2011; Standfuss et al., 2011; Deupi et al., 2012; Singhal et al., 2013). These structures have revealed the importance of ICL2 and ICL3 for GPCR interactions with the Gα subunit of the heterotrimeric G proteins (Rasmussen et al., 2011; Ma et al., 2014; Moreira, 2014). Although family B GPCRs share little sequence similarity with family A GPCRs in the three cytoplasmic loops, mutagenesis studies showed the important role of ICL3 in the selectivity and efficiency of G-protein coupling and activation (Cypess et al., 1999; Hallbrink et al., 2001; Bavec et al., 2003; Conner et al., 2006), as detailed below.

### Interactions and structural changes

Although intracellular loops have been suggested essential for G protein coupling for family B GPCRs, the specific interactions that govern the coupling between GPCRs and G proteins vary from receptor to receptor. Most family B GPCRs can recognize multiple G proteins, such as G_s_, G_q∕11_, and G_i∕o_, increasing the complexity of the signaling processes (Rashid et al., 2004). Here, we will discuss the key interactions involved in G protein recognition for the most heavily investigated family B GPCR, GLP-1R, followed by a summary of the studies for other members in family B.

Studies of GLP-1R reveal that the coupling of various G proteins mainly takes place in ICL3 while ICL1 plays a supplementary role (Figure 12, top). A deletion in ICL3 reveals that a key block, Lys334-Leu335-Lys336, in the N-terminal region of this loop is required for coupling to G_s_ (Figure 12, top left; Takhar et al., 1996). Moreover, Hallbrink et al. used peptides to mimic different parts of ICL3 in GLP-1R and concluded that the N-terminal part of this loop exclusively stimulates G_s_ and the C-terminal part exclusively stimulates G_i_ (Figure 12, top; Hallbrink et al., 2001). Further studies found mutating three other amino acids, Val327, Ile328, and Val331, significantly decrease G_s_ activation, further corroborating the data showing that the N-terminal region of ICL3 is involved in G_s_ activation (Mathi et al., 1997). In addition to ICL3, ICL1 has also been shown to participate in G_s_ coupling, as a single-point mutation in this region (R176A) is sufficient to significantly reduce cAMP production (Figure 12, top left; Mathi et al., 1997). ICL1 may also play an essential role in G_i_ and G_o_ coupling. Studies using a chimeric rhodopsin receptor with ICL1 of GLP-1R in the place of rhodopsin's ICL3 show the receptor signals through G_i_ and G_o_ by coupling with the GLP-1R ICL1 rather than G_t_, the transducin G protein specifically coupled to rhodopsin (Yamashita et al., 2008). Bavec and coworkers performed a study to explore how peptides derived from the first, second and third intracellular loops of GLP-1R differentiate the G protein signaling pathways. The results suggest that ICL3 is the major determinant for binding to all G proteins (G_s_, G_o_, G_i_, and G_11_), whereas the other two intracellular loops are important in the selectivity of G proteins via modulating the interactions between ICL3 and G proteins (Figure 12, top; Bavec et al., 2003).

Studies of other family B GPCRs, including PTH1R, SCTR, and CGRP receptor, reach similar conclusions that ICL3 is the key to G protein coupling while ICL1 and ICL2 may also play subsidiary roles (Figure 12). For PTH1R, the N-terminal region of ICL3 has been suggested to interact with G_s_ and G_q_ (Figure 12, middle left; Huang et al., 1996) and the C-terminal region of ICL2, especially the EKKY segment, has been proven important for G_q_ coupling (Iidaklein et al., 1997). For SCTR, mutation studies confirm that at least one residue in ICL1 is involved in G_q_ signaling, and that the N-terminal and C-terminal regions of ICL3 account for G_s_ and G_q_ coupling, respectively (Garcia et al., 2012). For CGRP receptor, mutagenesis studies show that ICL1 is important for G_s_ coupling and that the N-terminal region of ICL3 is a key G_s_ binding motif, while the C-terminal region of ICL3 impairs G_s_ binding (Figure 12, bottom left; Conner et al., 2006). For VPAC_1_R, ICL2 has no observable effect on G_s_ coupling, but Lys322 on ICL3 together with another charged residue (Glu394) on the proximal C-terminal tail of the receptor, play key roles in coupling with G_s_ (Figure 12, bottom middle; Langer et al., 2002). For GCGR, replacement of all or selected intracellular loops with the ICL1 on D4 dopamine receptor, a family A GPCR, suggests that ICL2 and ICL3 are required for G_s_ and G_q_ coupling (Figure 12, middle right; Cypess et al., 1999).

As summarized in Figure 12, the general conclusion is that ICL3 is the key determinant for family B GPCRs to activate G proteins of all types, including G_s_, G_q_, G_i∕o_, and G_11_. The specific regions within each receptor that are responsible for selecting G proteins remain unclear, although accumulating evidence suggests that the N-terminal portion within ICL3 is essential for G_s_ coupling and in some receptors for G_q_ coupling as well. However, the selectivity of G proteins can also depend on the binding of receptors to various ligands and accessory proteins. Thus, the molecular mechanism behind the selectivity and activation of G proteins for family B GPCRs is a profound research topic that requires further exploration.

## Interaction with accessory proteins

Aside from G proteins, GPCRs can also associate with a variety of accessory proteins that play important roles in modulating receptor functions, including cell surface expression of receptors, selectivity of hormone ligands, and selection of G protein signaling pathways (as reviewed in Couvineau and Laburthe, 2012). These accessory proteins are diverse, including but not limited to receptor-activity-modifying proteins (RAMPs), PDZ domain-containing proteins, cytoskeleton proteins, chaperone molecules, and kinases (Couvineau and Laburthe, 2012). In this section, we will review three representative accessory proteins: RAMPs, calmodulin (CaM), and Na/H exchange regulatory factors (NHERFs). We will focus on their role in the signaling process and the specific sequence motifs responsible for receptor interactions.

**Figure 13:**
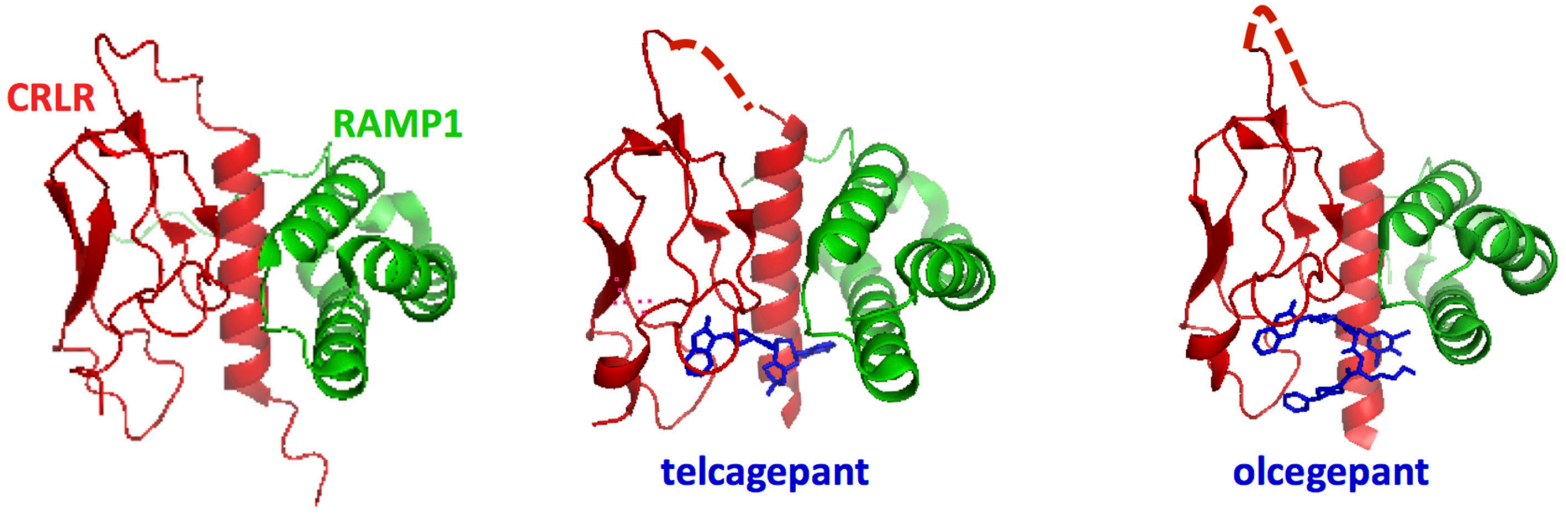
**Ligand binding sites in calcitonin receptors likely involve both the receptor N-terminal domain and RAMPs**. The crystal structures for the calcitonin gene related peptide receptor in complex with two small molecule antagonists (blue). The binding pocket for the small molecules is formed by the interface residues in the two components of the heterodimer: calcitonin receptor-like receptor (CRLR, red) and RAMP1 (ter Haar et al., 2010).

Among the accessory proteins that interact with family B GPCRs, the RAMP family is the most extensively studied (Parameswaran and Spielman, 2006). They have been shown to dimerize with family B GPCRs, including the calcitonin receptors, PTH1R, and VPAC_1_R (Christopoulos et al., 2003; Archbold et al., 2011). There are three isoforms of RAMPs (RAMP 1, 2, and 3), which all have a single transmembrane domain and an 80-amino acid N-terminal domain folded into three helices (Figures 3, 13; Parameswaran and Spielman, 2006). The binding interface between RAMPs and family B GPCRs has not been completely identified. The crystal structure of the CGRP receptor, a heterodimer of CRLR and RAMP1, shows the binding interface between N-terminal domains in the extracellular region of RAMP1 and receptor (Figure 13; ter Haar et al., 2010), where hydrogen-bonding, hydrophobic, and electrostatic interactions are key interactions. However, no structural information is available for interactions between RAMP and the transmembrane domain or the cytoplasmic domain.

The binding of different RAMP isoforms to family B receptors can modulate at least three functions of family B GPCRs. First, the binding of RAMPs regulates receptor trafficking. For example, the calcitonin receptor (CTR) changes its cell surface expression upon binding to RAMPs (McLatchie et al., 1998). Second, the binding of RAMPs can change the G protein coupling profile. For example, RAMP2 association with VPAC_1_R increases G_q_ coupling without affecting the G_s_ coupling (Christopoulos et al., 2003). Finally, RAMPs can switch selectivity of ligands. For instance, CRLR binds CGRP, amylin peptide 1, and amylin peptide 2 after interacting with RAMP1, 2, and 3, respectively (Parameswaran and Spielman, 2006). The mechanism of how RAMPs can bias the ligand binding is not fully understood; however, several mutagenesis studies of RAMPs suggested that RAMPs are likely to directly participate in ligand-GPCR interaction (Qi and Hay, 2010; Archbold et al., 2011). Nonetheless, it is still unclear at the molecular level how receptor-RAMP interactions, especially in the transmembrane and cytoplasmic regions, can modulate the above functions of family B GPCRs.

Aside from RAMP, another accessary protein, calmodulin, also interacts with a number of family B GPCRs (Mahon and Shimada, 2005). Calmodulin is a cytosolic calcium-sensing protein. It interacts with receptors in a Ca^2+^-dependent manner through a consensus CaM-binding motif in the receptors' C-terminal tails, which is called the basic 1-5-8-14 domain (Mahon and Shimada, 2005). This binding motif is found in PTH1R, VPAC1_1_R, PAC1R, CRF1R, CTR, GLP-1R, GLP-2R, SCTR, and GHRHR (Mahon and Shimada, 2005). Except SCTR and GHRHR, all these receptors have been shown to bind to CaM. While Mahon and Shimada showed that the binding of CaM to PTH1R decreases activation of G_q_ (Mahon and Shimada, 2005), the biological functions of CaM still largely remain unknown.

Another important family of accessory proteins is the four members of the NHERFs (Ardura and Friedman, 2011). These proteins are present in the cytoplasm and contain a C-terminal ezrin-radixin-moesin (ERM)-binding domain and two tandem PDZ domains (Ardura and Friedman, 2011). The ERM-binding domain is known to bind ERM proteins, mediating interactions with actin filaments while the PDZ domain is a common protein interaction module composed of 80–90 amino-acids found in signaling proteins that binds to short amino acid motifs of the C-terminal tail of target proteins (Ardura and Friedman, 2011). The role of PDZ domains of NHERFs is more extensively studied. This domain recognizes two sequences, of which class I is important in interactions with family B GPCRs (Ardura and Friedman, 2011). The class I sequence, ETVM, in the C-terminus of PTH1R facilitates interactions of the receptor with NHERFs (1 and 2) (Mahon et al., 2002; Mahon and Segre, 2004). The PTH1R-NHERF interaction not only leads to recruitment of multiple downstream signaling effectors, but also switches the selectivity of G protein from G_s_ to G_q_ (Mahon et al., 2002; Wang et al., 2010). Sequence analysis of family B GPCRs suggests the presence of class I PDZ binding motifs in several other members, including VPAC_2_R, SCTR, GLP-2R, and CRF1R (Couvineau and Laburthe, 2012). However, whether or not these receptors interact with NHERFs still needs to be examined.

Aside from the above accessory proteins, many other accessory proteins also interact with family B GPCRs, including calpain and the 14-3-3 protein, adding complexity to the signal transduction process of family B GPCRs (Couvineau and Laburthe, 2012). Indeed, identification of accessory proteins and investigation of their functional roles is still at the infant stage. Continuous efforts are necessary to explore the roles of these accessory proteins in the signaling process of family B GPCRs.

## Challenges and outlook

To summarize, this review discusses the current understanding of the activation mechanism of family B GPCRs focusing on three steps: (1) ligand binding, (2) transmembrane conformational changes, and (3) G protein coupling. It also discusses how some accessory proteins may modulate activation. While the first step of ligand binding is relatively well understood and described by the two-domain ligand-binding model, the molecular mechanism of transmembrane conformational changes, and G protein coupling have remained largely unknown. Added to this is the crosstalk among ligands, G proteins, and accessory proteins with a family B GPCR, which tunes the structure and dynamics of the receptor and thereby changes the affinity toward different ligands and activates different isoforms of G proteins. All these add complexity to the signaling process of family B GPCRs, making mechanistic studies challenging.

A more complete understanding of the activation mechanism of family B GPCRs is an urgent need. As shown by mechanistic studies of family A GPCRs, biophysical characterizations can be useful: X-ray crystallography (Palczewski et al., 2000; Jaakola et al., 2008; Choe et al., 2011; Rasmussen et al., 2011; Warne et al., 2011) and NMR (Ahuja et al., 2009; Smith, 2010) can determine structures of both inactive and active states, electron paramagnetic resonance can map patterns of helix rearrangement during activation (Altenbach et al., 2008), and other spectroscopic methods, such as fluorescence (Dunham and Farrens, 1999; Yao et al., 2006), Raman (Pan et al., 2002) and Fourier-transformed infrared (Fahmy et al., 2000; Mahalingam et al., 2008) can reveal structures and dynamics of receptors.

Nonetheless, these biophysical characterizations require purification of full-length, functional family B GPCRs in a sufficient amount. Many attempts have been made, ranging from purification from natural sources to heterologous expression systems, such as *E. coli*, mammalian cells, insect cells, and cell-free synthesis systems. So far, only three out of the many studies have shown functional, purified receptors can bind to the ligand and activate G proteins (Ohtaki et al., 1998; Shimada et al., 2002; Mitra et al., 2013). In addition, few studies perform biophysical studies on purified receptors to understand the conformational and dynamic changes during activation.

Recently, our laboratory developed a purification method for family B GPCRs, which directly purifies GPCRs into nanodiscs—disc-shaped particles containing a single layer of lipid bilayer that is stabilized by two membrane scaffold proteins (Mitra et al., 2013). This method reduces the contact of a receptor with detergent and eliminates the step of reconstitution of receptor from detergent solubilized environment into lipid bilayers. The receptor then has both the extracellular and cytoplasmic domains exposed to solution, enabling assessment of molecular interactions from both sides (Leitz et al., 2006; Banerjee et al., 2008; El Moustaine et al., 2012; Inagaki et al., 2012). Unlike detergent systems, nanodiscs can stabilize receptors without the presence of extra micelles that results from maintaining a detergent concentration above the critical micelles concentration (CMC). Thus, the use of nanodiscs in stabilization of receptors can reduce backgrounds in biophysical measurements, such as light scattering in optical spectroscopy or detergent signals in NMR experiments. Hence, the nanodisc system can provide flexibility in designing biophysical experiments (Serebryany et al., 2012), highlighting it as an ideal platform for molecular biophysical characterizations.

Aside from structure-function correlation, the dynamics of the molecular mechanism of family B GPCRs remain largely unknown and unexplored. While solution NMR and single-molecule spectroscopy are commonly used to study protein dynamics, they are not commonly applied to GPCRs. The advancement in computational biology and molecular modeling is deepening our understanding of molecular dynamics (Singh et al., 2015). However, this requires building reliable models for full length family B GPCRs, which is challenging. Even though the transmembrane domain structures of two family B receptors became available, determining the relative orientations of the N-terminal domain and the transmembrane domain remains a difficult task, which requires a synergistic experimental and computational approach. A few groups are working toward building computational models of full-length family B GPCRs, specifically for SCTR, GLP-1R, and PTH1R (Thomas et al., 2008; Coopman et al., 2011; Dong et al., 2011a; Kirkpatrick et al., 2012). These models still require experimental studies of full-length receptors for verification and refinement.

In addition, development of drugs targeting family B GPCRs is still challenging in at least two ways: (1) peptide ligand derivatives as drugs suffer from rapid renal clearance and proteolytic degradation; (2) small molecules as drugs are hard to identify due to limited structural information. There are thus considerable efforts drawn to improve the efficacy of peptide-based drugs (Zuckermann and Kodadek, 2009; Denton et al., 2013; Johnson et al., 2014; Liu et al., 2015); and the current advances in structural biology, especially the crystal structure of small-molecule docked CRF1R's transmembrane, will shed light on rational design of small molecules targeting family B GPCRs.

A theme that runs through this review is the complexity of the signaling processes of family B GPCRs, which comes from modulations of family B GPCRs by ligands, G proteins, and accessory proteins. These modulations change the selectivity of the receptors during ligand sensing and G protein coupling, greatly enriching the information content of cellular communication with the environment and the cellular response to the hormone signaling processes. The field of family B GPCRs is beginning to understand that the signaling process is not only through interactions of a receptor with a specific pair of a ligand and a G protein but also global changes in receptor structure and dynamics regulated by simultaneous and/or sequential interactions with ligand, G proteins, and accessory proteins. A complete understanding of the molecular mechanism of GPCR activation will continue to require a collaborative effort, from high-resolution structural determinations and quantitative biophysical characterizations, to cellular studies of signaling processes and computational molecular modeling, in order to reveal the underlying activation mechanism of family B GPCRs at the fundamental level.

### Conflict of interest statement

The authors declare that the research was conducted in the absence of any commercial or financial relationships that could be construed as a potential conflict of interest.
