# Supplementary material for: Transmembrane signal transduction by peptide hormones via family B G protein-coupled receptors
Source: Front Pharmacol. 2015 Nov 5;6:264. doi: 10.3389/fphar.2015.00264 (PMC4633518; doi:10.3389/fphar.2015.00264)
Supplement: Supplementary file 1 [file Table1.DOCX]

Table 1: Experimental details from experiments on Family B GPCRs cited in the text, sorted by domain.

| **Receptor** | **Residue(s)** | **Experiment** | **Study Details** | **Results** | **Citation** |
| --- | --- | --- | --- | --- | --- |
| **Extracellular domain** | | | | | |
| CLR | I41A  A44L  Q45A  C48A  Y49A | Radioligand binding  cAMP accumulation | Alanine scanning; competitive binding | Decreased binding affinity and cAMP production of CGRP | (Barwell et al., 2010) |
|  | A273L  R274A  D280A  W283A  I284A  S285A  D288A  Y277A  Y278A  C282A  D287A  L290A  L291A | cAMP accumulation | Alanine scanning of EL2 | Reduced CGRP and AM potency  Reduced CGRP potency | (Woolley et al., 2013) |
| CRF1R | W259A  F260A | Radioligand binding  cAMP accumulation | Alanine scanning of EL2 residues;  Competitive binding assays, membrane preparations | Reduce binding affinity and potency of sauvagine and CRF | (Gkountelias et al., 2009) |
| CRF2Rβ | R112E  I67E | Radioligand binding | Competitive binding assay of CRF2B in membrane preps | 7 fold decrease in asstressin binding  100 fold decrease in astressin binding | (Grace et al., 2004) |
| GLP1R | E127A  L32A  T35 A  P90A  R121A | Radioligand binding  cAMP accumulation | Competitive binding assay to GLP1R membrane preparations; cell based cAMP assays | 7 fold decrease in Ex-4 affinity, no change in GLP-1 affinity  Decrease in Ex-4 potency and affinity; no change in GLP-1  Decrease in Ex-4 potency (cAMP production)  Decrease in GLP-1 and Ex-4 affinity | (Underwood et al., 2010) |
|  | C229A  C296A  C229A/  C296A | Radioligand binding  cAMP accumulation | Competitive binding assays | Decrease in GLP-1 binding affinity;  C229A greatly reduced GLP-1 potency but double mutant does not affect potency | (Mann et al., 2010) |
| Glucagon | N-terminal domain 126-137  EL1 206-219 | cAMP accumulation  radioligand binding | Chimeric receptor construct with SCTR or GLP1R | Residues 126-137 and 220-231 implicated in ligand binding | (Unson et al., 2002) |
|  | Y65A  Q113E | Luciferase assays | Cell based reporter assays | 10 fold increase  4 fold increase | (Koth et al., 2012) |
| PACAPR | E104R  E117R  Y118A | Fluorescence polarization anisotropy | PACAP peptide binding PACAPR N-terminal domain | Around 5 fold decrease in binding affinity | (Sun et al., 2007) |
| PTH1R/CT | N-terminal domain | cAMP accumulation  radioligand binding | Chimeric PTH1R/CTR constructs with N-terminal domains exchanged | Receptor ligand binding and activity maintained when N-terminal domain of the receptor and C-terminus of the ligand correspond | (Bergwitz et al., 1996) |
| PTH1R | Y421C  F424C  L368C | Disulfide trapping cross-linking | Radiolabeled Cys-PTH peptide added to PTH1R transfected cells; crosslinking shown on SDS-PAGE gels run with or without reducing agent | N terminus of PTH is close to these 3 residues | (Monaghan et al., 2008) |
| VPAC_1_R | D68G  W73G  G109 | Radioligand binding | Competitive ligand binding assay with cell membranes with VPAC_1_R | No ligand binding obsereved | (Couvineau et al., 1995) |
| **TM domain** | | | | | |
| GLP1R | Y152A  R190A  Y235A  H363A  E364A;  K197A  Q234A  W284A  D198A  R310A  K197A  W284A  R310A  H363A | Radioligand binding  cAMP accumulation | Ala-mutations | Reduced binding affinity for GLP-1 or/ and exendin  Uncoupled from cAMP generation | (K. Coopman, 2011) |
|  | K197A  D215A  R190A  N240A | Radioligand binding  cAMP accumulation | Ala-mutations | Reduced binding affinity  Low cAMP generation | (Xiao et al., 2000) |
| VPAC_1_R | K127 | Photocrosslinking ligand binding  cAMP accumulation | Photoaffinity labeling | K127 directly interacts with VIP ligand | (Ceraudo et al., 2008) |
|  | R188Q  R188L  K195Q  K195I | Radioligand binding  cAMP accumulation | Point mutations | R188 and K195 are important for VIP recognition | (Solano et al., 2001) |
|  | K143A  T144A  T147A  L375A | Photocrosslinking ligand binding | Ala- mutations  Photoaffinity labeling | Reduced binding affinity | (Ceraudo et al., 2012) |
| CRF1R | M305 mutants | Radioligand binding | Point mutations | M305I reduces binding affinity of non-peptide antagonists | (Hoare et al., 2004) |
| CGRP | L195A  V198A  A199L  H219A  L220A  L222A | Radioligand binding  cAMP accumulation | Ala- mutations | Low cAMP generation  Reduced αCGRP binding | (Barwell et al., 2011) |
| Secretin | TM1  Y124A  Y124H  Y128A  Y128H | cAMP accumulation | Point mutations | Y128 directly interact with Asp3 of secretin | (Di Paolo et al., 1998b) |
|  | TM2  D174  K173  R166 | cAMP accumulation | Point mutations | R166 is the major determinant for interaction with Asp3 of secretin | (Di Paolo et al., 1998a) |
| PTH1R | TM2  A234A  V235A  K240A | Radioligand binding, Photochemical cross-linking | Ala- mutations | A234, V235 and K240A affect photo-insertion efficiency, and K240 also interact with ligand residues directly | (Gensure et al., 2003) |
| GCGR | Chimeric TM2  I194K  D385E | Radioligand binding  cAMP accumulation | Point mutations, chimeric receptor constructs with GLP1R | I194 (TM2) and D385 (TM7) are important for ligand selectivity | (Runge et al., 2003) |
|  | I194K | Radioligand binding  cAMP accumulation | Point mutations | Reduced affinity and potency | (Perret et al., 2002) |
| GIPR | R183A  R190A  R300A  F357A  Q224A | cAMP accumulation | Ala-mutations | R183, R190, R300 and F357 mutations caused reduced cAMP generation;  R183, Q224, R300 and F357 interact with GIP | (Yaqub et al., 2010) |
|  | T340P | cAMP accumulation | Point mutation | Point mutation of T340P results in constitutive activation. | (Tseng and Lin, 1997) |
| **Cytoplasmic domain** | | | | | |
| PTH1R | V378A L379A  T381A  K382A | radioligand binding  cAMP accumulation  IP production | Ala-mutations | V378A and L379A: reduced IP but normal cAMP response  T381A: reduced cAMP but normal IP response  K382A: reduced cAMP and IP responses | (Huang et al., 1996) |
|  | EKKY (317-320) | radioligand binding  cAMP accumulation  IP production | EKKY replaced with DSEL | No PLC activity (G_q_) but full cAMP activity | (Iida-Klein et al., 1997) |
| Glucagon | ICL2  ICL3 | radioligand binding  cAMP accumulation  IP production | Replacement of ICLs using the ICL1 of the D4 dopamine receptor | Replacement of either loop: no cAMP activity  Replacement of both loops: no cAMP or IP activities | (Cypess et al., 1999) |
| GLP-1R | K334-L335-K336 | radioligand binding  cAMP accumulation | Block deletion | ~10 fold decrease in cAMP assay | (Takhar et al., 1996) |
|  | R176A  V327A  I328A V331A | radioligand binding  cAMP accumulation | Ala-mutations | Reduction in cAMP response | (Mathi et al., 1997) |
|  | ICL3-N terminal half  ICL3-C terminal half | GTPase activity  GTPγS binding  cAMP accumulation | Synthetic peptides representing the regions of interest for G protein stimulations | N-terminal part: exclusively stimulates cholera toxinsensitive  G proteins  C-terminal part: stimulates pertussis toxin-sensitive G-proteins | (Hällbrink et al., 2001) |
|  | ICL1  ICL2  ICL3 | G protein activation studies for G_s_, G_o_, G_i1_ and G_11_ | Synthetic peptides representing the regions of interest for G protein stimulations | ICL3: required for binding with all four G proteins  ICL1 and ICL2: activate G_s_, do not affect G_o_  ICL1: does not affect G_i1_ and G_11_  ICL2: actives G_i1_ and G_11_ | (Bavec et al., 2003) |
|  | ICL1 | G protein activation assay (radionucleotide filter-binding assay) | Replacement of ICL3 in rhodopsin by ICL1 in GLP-1R or SCTR | The mutant receptor responds to G_i_ and G_o_ but not G_t_ | (Yamashita et al., 2008) |
| SCTR | R153A  K302A/L303A  R318A/R321A |  | Ala-mutations | R153A: reduced Ca^2+^ but not cAMP response  K302A/L303A: reduced cAMP response  R318A/R321A: reduced cAMP and Ca^2+^ responses | (Garcia et al., 2012) |
| CGRP | L147A  R151A  I290A  L294A  R314A | radioligand binding  cAMP accumulation  receptor internalization | Ala-mutations | Reduction in cAMP response | (Conner et al., 2006) |
| VPAC_1_R/ VPAC_2_R | IRKS (328–331) in VPAC_1_R  VGGN (315-318) in VPAC_2_R | radioligand binding  cAMP accumulation  IP production | Chimeric receptors with the four amino acid segments swapped for each receptor | IRKS determines responses typical for VPAC_1_R  VGGN determines responses typical for VPAC_2_R | (Langer et al., 2002) |

**References**

Barwell, J., Conner, A., and Poyner, D.R. (2011). Extracellular loops 1 and 3 and their associated transmembrane regions of the calcitonin receptor-like receptor are needed for CGRP receptor function. *Biochimica et Biophysica Acta (BBA) - Molecular Cell Research* 1813**,** 1906-1916. doi: <http://dx.doi.org/10.1016/j.bbamcr.2011.06.005>.

Barwell, J., Miller, P.S., Donnelly, D., and Poyner, D.R. (2010). Mapping interaction sites within the N-terminus of the calcitonin gene-related peptide receptor; the role of residues 23–60 of the calcitonin receptor-like receptor. *Peptides* 31**,** 170-176. doi: <http://dx.doi.org/10.1016/j.peptides.2009.10.021>.

Bavec, A., Hällbrink, M., Langel, Ü., and Zorko, M. (2003). Different role of intracellular loops of glucagon-like peptide-1 receptor in G-protein coupling. *Regulatory Peptides* 111**,** 137-144. doi: <http://dx.doi.org/10.1016/S0167-0115(02)00282-3>.

Bergwitz, C., Gardella, T.J., Flannery, M.R., Potts, J.T., Kronenberg, H.M., Goldring, S.R., and Jüppner, H. (1996). Full Activation of Chimeric Receptors by Hybrids between Parathyroid Hormone and Calcitonin: EVIDENCE FOR A COMMON PATTERN OF LIGAND-RECEPTOR INTERACTION. *Journal of Biological Chemistry* 271**,** 26469-26472. doi: 10.1074/jbc.271.43.26469.

Ceraudo, E., Hierso, R., Tan, Y.-V., Murail, S., Rouyer-Fessard, C., Nicole, P., Robert, J.-C., Jamin, N., Neumann, J.-M., and Robberecht, P. (2012). Spatial proximity between the VPAC1 receptor and the amino terminus of agonist and antagonist peptides reveals distinct sites of interaction. *The FASEB Journal* 26**,** 2060-2071.

Ceraudo, E., Murail, S., Tan, Y.-V., Lacapere, J.-J., Neumann, J.-M., Couvineau, A., and Laburthe, M. (2008). The vasoactive intestinal peptide (VIP) α-Helix up to C terminus interacts with the N-terminal ectodomain of the human VIP/pituitary adenylate cyclase-activating peptide 1 receptor: photoaffinity, molecular modeling, and dynamics. *Molecular Endocrinology* 22**,** 147-155.

Conner, A.C., Simms, J., Howitt, S.G., Wheatley, M., and Poyner, D.R. (2006). The second intracellular loop of the calcitonin gene-related peptide receptor provides molecular determinants for signal transduction and cell surface expression. *Journal of Biological Chemistry* 281**,** 1644-1651.

Couvineau, A., Gaudin, P., Maoret, J.J., Rouyerfessard, C., Nicole, P., and Laburthe, M. (1995). Highly Conserved Aspartate 68, Tryptophane 73 and Glycine 109 in the N-Terminal Extracellular Domain of the Human VIP Receptor Are Essential for Its Ability to Bind VIP. *Biochemical and Biophysical Research Communications* 206**,** 246-252. doi: <http://dx.doi.org/10.1006/bbrc.1995.1034>.

Cypess, A.M., Unson, C.G., Wu, C.-R., and Sakmar, T.P. (1999). Two cytoplasmic loops of the glucagon receptor are required to elevate cAMP or intracellular calcium. *Journal of Biological Chemistry* 274**,** 19455-19464.

Di Paolo, E., De Neef, P., Moguilevsky, N., Petry, H., Bollen, A., Waelbroeck, M., and Robberecht, P. (1998a). Contribution of the second transmembrane helix of the secretin receptor to the positioning of secretin. *FEBS letters* 424**,** 207-210.

Di Paolo, E., Petry, H., Moguilevsky, N., Bollen, A., De Neef, P., Waelbroeck, M., and Robberecht, P. (1998b). Mutations of aromatic residues in the first transmembrane helix impair signalling by the secretin receptor. *Receptors & Channels* 6**,** 309-315.

Garcia, G.L., Dong, M., and Miller, L.J. (2012). Differential determinants for coupling of distinct G proteins with the class B secretin receptor*. American Journal of Physiology-Cell Physiology* 302**,** C1202-C1212. doi: DOI 10.1152/ajpcell.00273.2011.

Gensure, R.C., Shimizu, N., Tsang, J., and Gardella, T.J. (2003). Identification of a contact site for residue 19 of parathyroid hormone (PTH) and PTH-related protein analogs in transmembrane domain two of the type 1 PTH receptor. *Molecular Endocrinology* 17**,** 2647-2658.

Gkountelias, K., Tselios, T., Venihaki, M., Deraos, G., Lazaridis, I., Rassouli, O., Gravanis, A., and Liapakis, G. (2009). Alanine Scanning Mutagenesis of the Second Extracellular Loop of Type 1 Corticotropin-Releasing Factor Receptor Revealed Residues Critical for Peptide Binding. *Molecular Pharmacology* 75**,** 793-800. doi: 10.1124/mol.108.052423.

Grace, C.R.R., Perrin, M.H., Digruccio, M.R., Miller, C.L., Rivier, J.E., Vale, W.W., and Riek, R. (2004). NMR structure and peptide hormone binding site of the first extracellular domain of a type B1 G protein-coupled receptor. *Proceedings of the National Academy of Sciences of the United States of America* 101**,** 12836-12841. doi: 10.1073/pnas.0404702101.

Hällbrink, M., Holmqvist, T., Olsson, M., Östenson, C.-G., Efendic, S., and Langel, Ü. (2001). Different domains in the third intracellular loop of the GLP-1 receptor are responsible for Gαs and Gαi/Gαo activation. *Biochimica et Biophysica Acta (BBA) - Protein Structure and Molecular Enzymology* 1546**,** 79-86. doi: <http://dx.doi.org/10.1016/S0167-4838(00)00270-3>.

Hoare, S.R.J., Sullivan, S.K., Schwarz, D.A., Ling, N., Vale, W.W., Crowe, P.D., and Grigoriadis, D.E. (2004). Ligand Affinity for Amino-Terminal and Juxtamembrane Domains of the Corticotropin Releasing Factor Type I Receptor:  Regulation by G-Protein and Nonpeptide Antagonists. *Biochemistry* 43**,** 3996-4011. doi: 10.1021/bi036110a.

Huang, Z., Chen, Y., Pratt, S., Chen, T.-H., Bambino, T., Nissenson, R.A., and Shoback, D.M. (1996). The N-terminal region of the third intracellular loop of the parathyroid hormone (PTH)/PTH-related peptide receptor is critical for coupling to cAMP and inositol phosphate/Ca2+ signal transduction pathways. *Journal of Biological Chemistry* 271**,** 33382-33389.

Iida-Klein, A., Guo, J., Takemura, M., Drake, M.T., Potts, J.T., Abou-Samra, A., Bringhurst, F.R., and Segre, G.V. (1997). Mutations in the second cytoplasmic loop of the rat parathyroid hormone (PTH)/PTH-related protein receptor result in selective loss of PTH-stimulated phospholipase C activity. *Journal of Biological Chemistry* 272**,** 6882-6889.

K. Coopman, R.W., G. Robb, A. J. H. Brown, G. F. Wilkinson, D. Timms, G. B. Willars (2011). Residues within the Transmembrane Domain of the Glucagon-Like Peptide-1 Receptor Involved in Ligand Binding and Receptor Activation: Modelling the Ligand-Bound Receptor. *Molecular Endocrinology* 25**,** 1804-1818. doi: doi:10.1210/me.2011-1160.

Koth, C.M., Murray, J.M., Mukund, S., Madjidi, A., Minn, A., Clarke, H.J., Wong, T., Chiang, V., Luis, E., Estevez, A., Rondon, J., Zhang, Y., Hötzel, I., and Allan, B.B. (2012). Molecular basis for negative regulation of the glucagon receptor. *Proceedings of the National Academy of Sciences* 109**,** 14393-14398. doi: 10.1073/pnas.1206734109.

Langer, I., Vertongen, P., Perret, J., Waelbroeck, M., and Robberecht, P. (2002). A small sequence in the third intracellular loop of the VPAC1 receptor is responsible for its efficient coupling to the calcium effector. *Molecular Endocrinology* 16**,** 1089-1096.

Mann, R.J., Al-Sabah, S., De Maturana, R.L., Sinfield, J.K., and Donnelly, D. (2010). Functional coupling of Cys-226 and Cys-296 in the glucagon-like peptide-1 (GLP-1) receptor indicates a disulfide bond that is close to the activation pocket. *Peptides* 31**,** 2289-2293. doi: <http://dx.doi.org/10.1016/j.peptides.2010.09.015>.

Mathi, S.K., Chan, Y., Li, X., and Wheeler, M.B. (1997). Scanning of the glucagon-like peptide-1 receptor localizes G protein-activating determinants primarily to the N terminus of the third intracellular loop. *Molecular Endocrinology* 11**,** 424-432.

Monaghan, P., Thomas, B.E., Woznica, I., Wittelsberger, A., Mierke, D.F., and Rosenblatt, M. (2008). Mapping Peptide Hormone−Receptor Interactions Using a Disulfide-Trapping Approach†. *Biochemistry* 47**,** 5889-5895. doi: 10.1021/bi800122f.

Perret, J., Craenenbroeck, M., Langer, I., Vertongen, P., Grégoire, F., Robberecht, P., and Waelbroeck, M. (2002). Mutational analysis of the glucagon receptor: similarities with the vasoactive intestinal peptide (VIP)/pituitary adenylate cyclase-activating peptide (PACAP)/secretin receptors for recognition of the ligand’s third residue. *Biochemical Journal* 362**,** 389-394.

Runge, S., Gram, C., Bräuner-Osborne, H., Madsen, K., Knudsen, L.B., and Wulff, B.S. (2003). Three Distinct Epitopes on the Extracellular Face of the Glucagon Receptor Determine Specificity for the Glucagon Amino Terminus. *Journal of Biological Chemistry* 278**,** 28005-28010. doi: 10.1074/jbc.M301085200.

Solano, R.M., Langer, I., Perret, J., Vertongen, P., Juarranz, M.G., Robberecht, P., and Waelbroeck, M. (2001). Two Basic Residues of the h-VPAC1 Receptor Second Transmembrane Helix Are Essential for Ligand Binding and Signal Transduction. *Journal of Biological Chemistry* 276**,** 1084-1088. doi: 10.1074/jbc.M007696200.

Sun, C., Song, D., Davis-Taber, R.A., Barrett, L.W., Scott, V.E., Richardson, P.L., Pereda-Lopez, A., Uchic, M.E., Solomon, L.R., Lake, M.R., Walter, K.A., Hajduk, P.J., and Olejniczak, E.T. (2007). Solution structure and mutational analysis of pituitary adenylate cyclase-activating polypeptide binding to the extracellular domain of PAC1-RS. *Proceedings of the National Academy of Sciences* 104**,** 7875-7880. doi: 10.1073/pnas.0611397104.

Takhar, S., Gyomorey, S., Su, R.-C., Mathi, S.K., Li, X., and Wheeler, M.B. (1996). The third cytoplasmic domain of the GLP-1 [7-36 amide] receptor is required for coupling to the adenylyl cyclase system. *Endocrinology* 137**,** 2175-2178.

Tseng, C.-C., and Lin, L. (1997). A point mutation in the glucose-dependent insulinotropic peptide receptor confers constitutive activity. *Biochemical and Biophysical Research Communications* 232**,** 96-100.

Underwood, C.R., Garibay, P., Knudsen, L.B., Hastrup, S., Peters, G.H., Rudolph, R., and Reedtz-Runge, S. (2010). Crystal Structure of Glucagon-like Peptide-1 in Complex with the Extracellular Domain of the Glucagon-like Peptide-1 Receptor. *Journal of Biological Chemistry* 285**,** 723-730. doi: 10.1074/jbc.M109.033829.

Unson, C.G., Wu, C.-R., Jiang, Y., Yoo, B., Cheung, C., Sakmar, T.P., and Merrifield, R.B. (2002). Roles of Specific Extracellular Domains of the Glucagon Receptor in Ligand Binding and Signaling†. *Biochemistry* 41**,** 11795-11803. doi: 10.1021/bi025711j.

Woolley, M.J., Watkins, H.A., Taddese, B., Karakullukcu, Z.G., Barwell, J., Smith, K.J., Hay, D.L., Poyner, D.R., Reynolds, C.A., and Conner, A.C. (2013). The role of ECL2 in CGRP receptor activation: a combined modelling and experimental approach. *Journal of The Royal Society Interface* 10. doi: 10.1098/rsif.2013.0589.

Xiao, Q., Jeng, W., and Wheeler, M. (2000). Characterization of glucagon-like peptide-1 receptor-binding determinants. *Journal of Molecular Endocrinology* 25**,** 321-335.

Yamashita, T., Tose, K., and Shichida, Y. (2008). First Cytoplasmic Loop of Glucagon‐like Peptide‐1 Receptor Can Function at the Third Cytoplasmic Loop Position of Rhodopsin†. *Photochemistry and Photobiology* 84**,** 931-936.

Yaqub, T., Tikhonova, I.G., Lättig, J., Magnan, R., Laval, M., Escrieut, C., Boulègue, C., Hewage, C., and Fourmy, D. (2010). Identification of determinants of glucose-dependent insulinotropic polypeptide receptor that interact with N-terminal biologically active region of the natural ligand. *Molecular Pharmacology* 77**,** 547-558.
